# *C. elegans* Dopaminergic D2-Like Receptors Delimit Recurrent Cholinergic-Mediated Motor Programs during a Goal-Oriented Behavior

**DOI:** 10.1371/journal.pgen.1003015

**Published:** 2012-11-15

**Authors:** Paola Correa, Brigitte LeBoeuf, L. René García

**Affiliations:** 1 Department of Biology, Texas A&M University, College Station, Texas, United States of America; 2 Howard Hughes Medical Institute, College Station, Texas, United States of America; Stanford University School of Medicine, United States of America

## Abstract

C*aenorhabditis elegans* male copulation requires coordinated temporal-spatial execution of different motor outputs. During mating, a cloacal circuit consisting of cholinergic sensory-motor neurons and sex muscles maintains the male's position and executes copulatory spicule thrusts at his mate's vulva. However, distinct signaling mechanisms that delimit these behaviors to their proper context are unclear. We found that dopamine (DA) signaling directs copulatory spicule insertion attempts to the hermaphrodite vulva by dampening spurious stimulus-independent sex muscle contractions. From pharmacology and genetic analyses, DA antagonizes stimulatory ACh signaling via the D2-like receptors, DOP-2 and DOP-3, and Gα_o/i_ proteins, GOA-1 and GPA-7. Calcium imaging and optogenetics suggest that heightened DA-expressing ray neuron activities coincide with the cholinergic cloacal ganglia function during spicule insertion attempts. D2-like receptor signaling also attenuates the excitability of additional mating circuits to reduce the duration of mating attempts with unproductive and/or inappropriate partners. This suggests that, during wild-type mating, simultaneous DA-ACh signaling modulates the activity threshold of repetitive motor programs, thus confining the behavior to the proper situational context.

## Author Summary

An animal's behavior is a complex output displayed in response to diverse external cues, which are sensed and processed by the nervous system. Nerve cells translate sensory information into chemical secretions (neurotransmitters). These chemical signals allow neurons and muscles to communicate and coordinate motor responses. However, it is complicated how these signals are interpreted in neuronal circuits to start, continue, modify, and end specific behaviors, under the appropriate conditions. The neurotransmitter dopamine (DA) is involved in adjusting animal movements, thus DA neurotransmission is a candidate for coupling behaviors to the proper situational context. Here, we used *C. elegans* copulation to understand the DA-regulated neuronal mechanisms that promote when and where motor responses should be executed. During mating, DA is used as a feedback mechanism to adjust the activity of multiple sensory-motor neurons and muscles that promote the rhythmic thrusting of the male copulatory organs against his partner's vulval genitalia. If vulval signals are withdrawn when the male loses contact with his mate's genitalia, the DA-adjusted motor neurons' activities dampen to cease cue-independent genital penetration attempts. Therefore, DA secretions fine-tune these motor outputs to be exclusively displayed at the vulva and thus confine a behavior to its corresponding context.

## Introduction

Context-dependent motor patterns are the outcome of unique interplay amongst neuromodulators in the central nervous system (CNS). The neurotransmitter dopamine (DA) modulates gamma-aminobutyric acid (GABA), glutamate and acetylcholine (ACh) activity in cognitive and motor behaviors [1]–[6]. In vertebrates, DA adjusts motor outputs by selective synergy/antagonism of tiered neuronal population's activities [2], [7]. In the brain this regulation is initiated by DA secretion from the substantia nigra, which antagonizes post-synaptic cholinergic striatal interneurons [8]. The result is a context-dependent voluntary motion modulated by DA [9]–[11]. Disturbing the DA-ACh balance causes impulsive motor disorders as described in Parkinson's disease and choreas [12]–[15]. However, vertebrate and invertebrate models that fully encompass the *in vivo* cellular and molecular components of the DA-ACh interaction, which refine motor outputs, remain elusive.

With 383 neurons in males, the genetically tractable nematode *Caenorhabditis elegans* is a model for dissecting the cellular and molecular machinery involved in motor programs. DA secretion from sensory neurons mediates transitions between locomotor patterns to directly or indirectly regulate muscle contractile events [16]. Activation of D1-like G_αq_-coupled receptors has been shown to regulate forward-to-backward locomotor switches and swimming-to-crawling gaits [17]–[20]. The opposing signaling cascade, activating G_αo_-coupled D2-like receptors, reduces locomotion velocity upon finding novel food sources [21]–[23]. While these studies provide insight into general principles underlying DA neurotransmission, a more complex, goal-oriented and decision-based behavior such as male mating could better model subtle DA-ACh motor circuit regulation.

The *C. elegans* male mating circuit integrates sensory-motor cues that result in successful insertion of the copulatory spicules into the hermaphrodite vulva (Figure S1A) [24], [25]. The positioning of the male tail over the vulva is a stepwise process, redundantly executed by a bilateral set of nine sensory rays located at the male tail [26], [27]. When the male contacts a mate, putative mechano- and chemosensory neurons within each ray projection initiate backward scanning locomotion. Scanning behavior facilitates additional male-specific sensilla, located anteriorly and posteriorly of the cloacal region, to sense the hermaphrodite's vulva. Upon vulval contact, scanning behavior ceases and a subset of post-cloacal sensilla sensory-motor neurons, PCB and PCC, release ACh to promote spicule insertion attempts. Ionotropic and metabotropic ACh receptors located on multiple genital muscles induce the male to press his tail against the vulva and stimulate rhythmic movements of the attached copulatory spicules, so that they repetitively thrust against the vulval slit. Full insertion is achieved by additional ACh secretions from the putative proprioceptive SPC motor neurons. Simultaneous stimulation from the post-cloacal sensilla and SPC neurons mediates tonic muscle contraction, resulting in complete spicule protrusion from the tail [24], [25], [28]. Intrinsic and extrinsic factors likely modify the cholinergic circuit's activity prior to and during mating [29]–[33]. Male-specific dopaminergic motor-sensory neurons suggest that DA might modulate aspects of mating behavior. In this study, we use pharmacology, genetics, behavioral observation, calcium imaging and optogenetics to determine that DA signaling, partly through D2-like receptors down-modulates ACh signaling to restrict mating attempts to the vulva and from inappropriate mates.

## Results

### DA is required for efficient spicule insertion during mating

Male copulation requires monitoring mechanisms to initiate and terminate multiple sub-steps under the proper context. Mating begins when the male presses his tail against the hermaphrodite and moves backwards, scanning for the vulva [25], [34]. After he locates the vulva, he initiates repetitive 7-11 Hz spicule thrusts to breach the vulval slit. During this sub-behavior, the male progressively adopts an arched body posture, which persists throughout spicule insertion and sperm transfer (Figure S1A). In rare events, this arched posture is adopted during scanning. Successful ejaculation occurs after repeated attempts of these motor sub-behaviors [27], [28]. Molecules that promote mating execution have been identified, but few modulators that regulate and refine the behavior have been described [24], [26], [30], [35]–[41].

**Figure 1:**
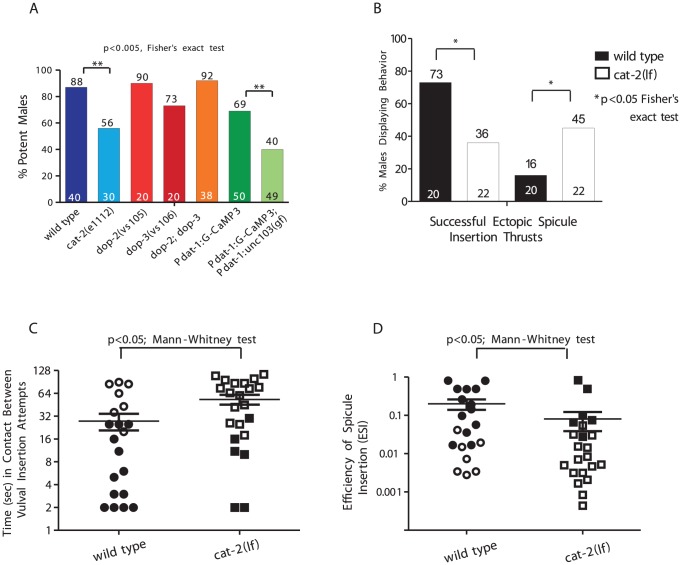
DA is required for spicule insertion during mating. The number of males tested and the percentage of potent males is listed at the bottom and top of the bars, respectively. (A) Mating potency. (B–D) Males were mated with paralyzed mates. (B) Percent of males displaying spicule related behaviors. (C) The duration in contact with a mate between insertion attempts. (D) The spicule efficiency index. Symbols represent an individual male performance. Open symbols represent unsuccessful insertions. Line and error bars represent mean and SEM.

DA signaling is known to modulate general C. *elegans* locomotor behaviors. Since 3 pairs of sex-specific sensory ray neurons secrete the neurotransmitter, DA is a candidate for modulating mating [16], [22]. Tyrosine hydroxylase is a key enzyme in the biosynthesis of DA. We first asked how well tyrosine hydroxylase deficient *cat-2(lf)* males mate [16], [22]. Initially we noticed that in *cat-2* male populations, a higher percentage displayed spontaneously protracted spicules (44%; n = 67) relative to wild type (10%; n = 62) *(p* = 0.0018, Fisher's exact test). This suggested that DA might down-modulate the spicule protraction circuit. When we paired a non-protracted virgin mutant or wild-type 1-day-old adult male with a 1-day-old moving hermaphrodite for 24 hrs, we found that 56% of *cat-2* males could sire progeny compared to the 88% of wild type (Figure 1A). To confirm that *cat-2* mating deficits were caused by DA depletion and not due to unknown background mutations, we attempted to phenocopy the *cat-2* behavioral defect in a different manner. Dopaminergic neurons were artificially hyperpolarized by expressing a hyperpolarizing UNC-103 ERG-like K+ channel (*unc-103(gf)*) [42] from the *dat-1* dopamine transporter promoter [43]. The *dat-1* promoter drives expression of this potassium channel exclusively in all DA neurons: CEP, ADE, PDE and rays 5,7,9A [26], [43]. Similar to *cat-2* mutants, males containing the *unc-103(gf)* transgene had increased number of spontaneously protracted spicules (28%; n = 46 vs. 4%; n = 27) (*p*-value = 0.04, Fisher's exact test), and had decreased ability to sire progeny (40%; n = 49 vs. 69%; n = 50) when compared to the transgenic control strain (Figure 1A). Since the behavior of *unc-103(gf)* transgenic males mimics the *cat-2* mating phenotype, this suggest that secretions from *dat-1* and *cat-2* expressing cells, likely DA, is necessary for efficient mating.

To ask how the *cat-2* mutation compromised mating, for 2 min we observed copulations between *cat-2* males and 2-day-old paralyzed hermaphrodites. We assayed mating initiation time, vulva contact duration and the number of vulva contacts (Figure S2), and found no difference between wild type and *cat-2* males. However, when we measured the average duration a male spent between vulval insertions attempts, we found that *cat-2* males had longer intervals than wild type (Figure 1B). This was because *cat-2* males displayed abnormal arched postures and precocious spicule thrusts at random areas on the hermaphrodite (Figure 1C). This defect also accounted for the mutant's reduced spicule penetration ability compared to wild type (36% vs. 73%, respectively). To quantify the variability of spicule insertion behavior, we calculated the efficiency of spicule insertion (E_SI_) in both groups. This metric combines how fast males initiate, sustain, re-attempt and complete spicule insertion. We found that *cat-2* males had a lower E_SI_ than wild type (0.19 vs. 0.075, Figure 1D). Thus, DA signaling promotes spicule insertion by lowering the probability of displaying non-productive ectopic spicule thrusts.

### Exposure to DA inhibits ACh-induced spicule protraction

We used pharmacology to address whether DA modulates the cholinergic spicule circuit by pre- or co-regulating the ACh response. ACh agonists artificially stimulate receptors on the spicule neurons and muscles to induce spicule protraction. These agonists include levamisole (LEV) and nicotine (NIC), which activate ionotropic ACh receptors (AChR), and oxotremorine-M (OXOM), which activates G_αq_ -coupled muscarinic AChRs (mAChR) [25], [28]. Arecoline (ARE) has been reported to stimulate mAChRs in the pharynx [44]; however, we found that in the spicule circuit, ARE is a non-selective agonist. For the spicule protraction circuit to be ARE-insensitive, a male must contain mutations in the NIC receptor (*acr-16 (ok789)*), LEV receptor (*unc-29(e193)*) and the OXOM receptor (*gar-3(gk305)*) (Table S1).

**Figure 2:**
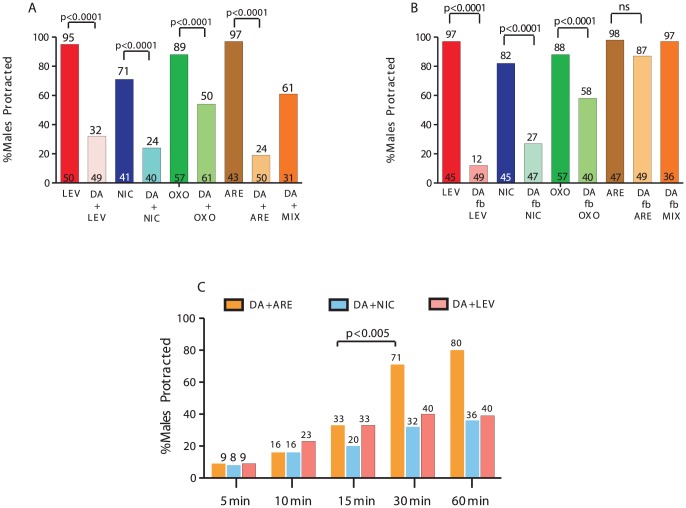
DA inhibits ACh-agonist-induced spicule protraction. The number of males tested and the percentage of spicule protracted males is listed at the bottom and top of the bars, respectively. (A&B) One-day-old virgin males were exposed to 30 mM DA and either LEV, NIC, OXOM or ARE. (A) DA simultaneous exposure with or (B) *followed by* (*fb*) ACh agonists. (C) Males were bathed in each drug combination for increasing duration. *p*-values determined with Fisher's exact test comparing mutants to wild type. MIX = LEV,NIC, OXO.

To test whether DA can attenuate the spicule protraction circuit, we exposed males to DA and ACh agonists simultaneously for 5 min and assayed males with protracted spicules. The effective concentrations inducing spicule protraction for 90% of the males (EC90) were 5 µM for LEV, 1 mM for both NIC and ARE and 50 mM for OXOM. The EC90 concentration for DA, inducing paralysis in 90% of the animals, was previously reported to be between 20–30 mM [21]. Therefore, we exposed one-day-old virgin males to 30 mM of DA combined with individual AChR agonists at their respective EC90 concentration (Figure 2A–2C). We found that DA reduced ACh-agonist induced protraction (Figure 2A), supporting our hypothesis that DA antagonizes ACh signaling.

To address if DA also preconditions the spicule protraction circuit to be less responsive to ACh stimulation, we bathed males in 30 mM of DA or water for 1 min, followed by (*f.b*) exposure to the EC90 ACh-agonist concentration. Exposure to DA or water did not induced spicule protraction in any male during a 2 min observation (Figure S1B). We found that DA pre-application still inhibited LEV- and NIC- and to a lesser extent OXOM-induced protraction (Figure 2B). Interestingly, DA pre-exposure didn't inhibit ARE-induced spicule protraction. To rule out the possibility that after DA exposure, ARE induces protraction independently of AChR stimulation, males were exposed to DA followed by an ACh-agonists mixture (MIX). This MIX contained LEV, NIC and OXOM at the EC90 concentrations. Similar to the ARE responses, we found that pre-exposure to DA did not inhibit the MIX-induced protraction, and when treating males with the MIX and DA simultaneously, spicule protraction was down-regulated (Figure 2A and 2B). These results suggest that DA down-modulation occurs simultaneously with ionotropic and muscarinic ACh signaling.

Since 1 min DA pre-exposure didn't antagonize ARE-induced protraction, we tested if the inhibition, which occurred with simultaneous exposure to both compounds, would also dissipate rapidly. Instead, we found that antagonism of sex muscle contractions dissipated by 30 min with DA+ARE treatment; for other agonist combinations, the inhibition remained up to 1 hr (Figure 2C). These results suggest that simultaneous DA and ACh secretion down-regulates spicule circuit excitability for a limited period.

Since ARE's non- selectivity approximates more native ACh signaling (Table S1), DA+ARE co-treatment was used to characterize the mechanism of DA down-modulation. First, we tested males containing the G-protein coupled receptor loss-of-function (*lf*) mutations *dop-1(vs100), dop-2(vs105), dop-3(vs106)* or *dop-4(tm1392)* [17], [21]. In the *dop-1* and *dop-4* mutants DA suppressed ARE-induced protraction to wild type levels, in accordance with previous studies where these receptors are found to enhance cellular excitability via Gα_q_ pathways [21], [45]. However, in the *dop-2(lf)* or *dop-3(lf)* single mutants and *dop-2*; *dop-3* double mutants DA did not decrease ARE-induced protraction to wild type levels (Table 1). An additional candidate that could mediate DA signaling is the chloride ligand gated channel LGC-53 [46]. We measured the DA+ARE response of a loss-of-function mutant for this channel, *lgc-53(n4330)* and found that these mutants had wild type DA+ARE sensitivity (Table 1). Although our results indicate that the DOP-2 and DOP-3 receptors partially mediate the DA modulatory response, we cannot rule out that DOP-1, DOP-4, LGC-53 and other yet-to-be identified DA receptors might act in specific combinations and in other cells to further attenuate ACh-induced spicule protraction.

**Table 1:** Dopamine receptors DOP-2 and DOP-3 mediate DA inhibition of ARE-induced protraction.

Genotype	% Males protracted (n)			P-value Fisher's exact test
	ARE (1 mM)	DA(30 mM) +ARE(1 mM)	DA(20 mM) +ARE(1 mM)	
Wild type	94 (61)	14 (117)	ND	
*dop-1 (vs100)*	75 (24)	20 (25)	ND	NS*
*dop-2 (vs105)*	92 (50)	58 (101)	ND	P<0.0001*
*dop-3 (vs106)*	90 (30)	59 (32)	ND	P = 0.0006*
*dop-4 (tm1392)*	93 (31)	26 (26)	ND	NS*
*dop-2 ; dop-3*	95 (76)	54 (85)	63 (40)	P = 0.005*
*lgc-53 (n4330)*	72 (25)	ND	16 (25)	NS*
*dop-2 ; pha-1*	95 (20)	ND	48 (52)	
*dop-3 ; pha-1*	100 (19)	ND	47 (40)	
*dop-2 rg*Ex462 [P*aex-3:dop-2*::CFP]	86 (30)	ND	43 (48)	NS**
*dop-2 rg*Ex467 [P*unc-103E:dop-2*::CFP]	86 (30)	ND	15 (51)	P = 0.0002**
*dop-3 rg*Ex490 [P*aex-3:dop-3*::YFP]	95 (20)	ND	24 (37)	NS**
*dop-3 rg*Ex482 [P*unc-103E:dop-3*::YFP]	90 (20)	ND	7 (39)	P = 0.001**

* Compared with wild-type DA+ARE sensitivity.

** Compared with mutants DA+ARE sensitivity in a *pha-1* background sensitivity.

All transgenic animals contain *pha-1(lf)*.

Since these D2-like receptors signal via Gα_o/i_ –pathways, the alleles *goa-1(n363), gpa-7(pk610), gpa-14(pk347)* and *gpa-16(it143)*, which impair Gα_o/i_- like molecules were also tested [47]. In all the single mutant males treated with DA and ARE, spicule protraction was still inhibited. Thus suggesting that these molecules acted in a redundant manner as published in other pathways [48]. We therefore tested the DA/ARE sensitivity in different combinations of loss-of-function mutations in these Gα_o/i_- like molecules (Table 2).We found that the double mutant *goa-1(lf); gpa-7(lf)* males were insensitive to the DA inhibition (Table 2). This is consistent with DOP-2, DOP-3, GOA-1 and GPA-7 expression in the spicule associated muscles and the neurons that innervate them (Figure S3) [47]. In contrast, DOP-1 and DOP-4 are not expressed in these cells, but only in a few ray neurons (data not shown and in [20]). Additionally, when DOP-2 and DOP-3 were transgenically expressed pan-neuronally from the *aex-3* promoter or in sex-muscles from the *unc-103E* promoter, restored DA down-modulation in *dop-2* and *dop-3* males was observed with DOP-2 and DOP-3 sex muscles expression (Table 1). These data suggest that DA antagonizes ACh signaling via DOP-2 and DOP-3 coupled to GOA-1 and GPA-7.

**Table 2:** Effect of mutant Gα alleles on DA inhibition of ARE-induced protraction.

Genotype	% Males protracted (n)		P -value Fisher's exact test
	ARE (1 mM)	DA(20 mM)+ ARE(1 mM)	
Wild type	94 (51)	21 (112)	
*goa-1(n363)*	97 (20)	12 (41)	NS*
*gpa-7(pk610)*	90 (20)	5 (20)	NS*
*gpa-16 (it143)*	90 (30)	11 (30)	NS*
*gpa-14 (pk347)*	90 (21)	10 (48)	NS*
*goa-1 (n363); gpa-7(pk610)*	98 (20)	62 (50)	P<0.001*
*gpa-7 (pk610); gpa14(pk347)*	93 (20)	25 (30)	NS*
*goa-1; gpa-7; (RNAi gpa-16)*	91 (19)	60 (31)	NS**

* Compared to wild-type DA+ARE sensitivity.

** Compared to *goa-1; gpa-7* DA+ARE sensitivity.

### Dynamic changes in DA ray neuron activities during arched body postures during spicule insertion attempts

In the hermaphrodite, broad D2-like receptor expression indicates that humoral DA secretions might activate these receptors [20]–[22], [49]. The 3 sex-specific sensory dopaminergic ray neurons (left/right Rn5A, Rn7A and Rn9A) located in the male tail might provide humoral or synaptic DA necessary to antagonize ACh signaling [26]. These ray neurons synapse to other ray neurons, inter- and motor neurons, post-cloacal sensilla neurons (p.c.s.) and sex muscles (Male Wiring Project, http://worms.aecom.yu.edu/pages/male_wiring_project.htm, [50]).

**Figure 3:**
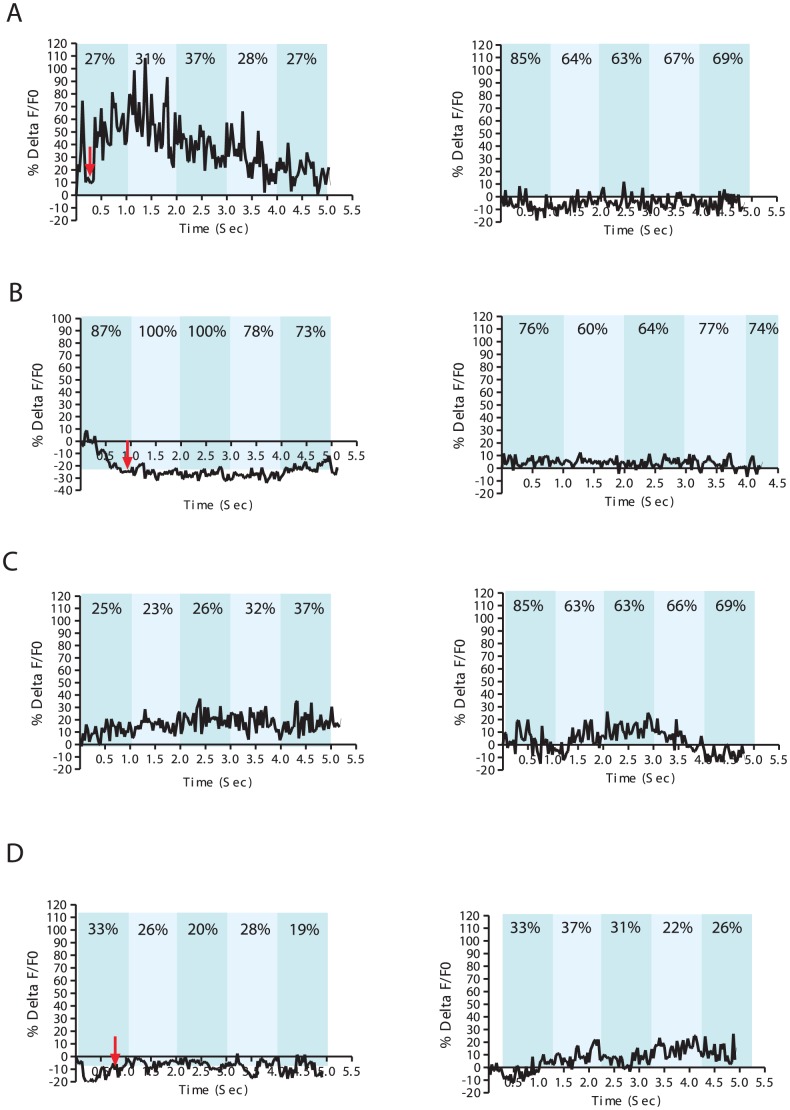
Ca^+2^ transients in DA ray neurons increase during arched postures. % ΔF/F0 trace for 5 seconds. Representative recordings for individual wild-type males with (A) an arched posture during spicule insertion attempts (left), (B) with non-arched posture during spicule insertion attempts (left), and (C) arched scanning posture (left). Non-arched scanning recordings for each male are displayed on the right (A–C). A *Pdat-1:unc-103(gf)* transgenic male displaying an arched posture at the vulva (left) and during scanning (right) (D). The In-Contact Length % (ICL%) are the numbers located at the top of each bar taken from a representative frame for each 1 sec intervals.

To measure the Ca^+2^ transients in DA ray neurons during mating, we compared the changes in fluorescence emissions of the G-CaMP Ca^+2^ sensor to a mDSred internal standard, both co-expressed from the DA reuptake transporter promoter (P*dat-1*). The G-CaMP transgene slightly reduces the mating potency of the males, but not statistically significant from wild-type males (Figure 1A). This indicates that the calcium binding property of the sensor does not interfere too greatly with dopaminergic cell function. To distinguish fluorescent changes caused by focusing artifacts when the male is performing scanning behavior, from fluorescent changes caused by neural activity, we imaged males in which Rn5,7,9A were additionally hyper-polarized via a *dat-1* promoter-expressing *unc-103(gf)* transgene. The mutant K+ channel should attenuate the ability of neurons to depolarize, and thus allow one to determine the fluorescence ranges that can confidently be attributed to cell activity. We found that throughout matings of *unc-103(gf)*-containing males, measurements in Rn5,7,9A fluorescence can range between 0 to a approximately 20% change (Figure 3D, Figure S4). These results suggest that focusing/motion artifacts can affect G-CaMP fluorescence measurements within this range.

In contrast, we sometimes observed 30–80% Ca^+2^ transient changes in the DA neurons when the male located the vulva (Figure 3A, Figure S4, Video S1). Occasionally, we also noticed up to 30% Ca^+2^ transient changes in DA ray neurons during *arch scanning* postures (Figure 3 and Figure S4). This observation led us to hypothesize that posture was correlated with DA ray neuron activity. To further correlate neuronal dynamics with copulatory postures, we measured In Contact Length percent (%ICL) between the male's body and the hermaphrodite, as a proxy for the adopted posture (arch vs. non-arch). The %ICL was higher when a male was engaged in non-arched vs. arched postures either during scanning or at the vulva (Figure 3). Lower %ICLs, indicating progressive arched postures, coincided with higher Ca^+2^ transient dynamics in DA ray neurons occurring while the male pressed his tail sternly against the vulva and with milder Ca^+2^ transient dynamics during scanning. However, if the male reached the vulva in a non-arched posture and proceeded to attempt spicule insertion in this posture, the observed Ca^+2^ changes were within baseline levels (10–20%) (Figure 3B, Figure 3S). This suggests that during arched postures, DA ray neurons might be more active to down-modulate possible spicule circuit cholinergic activity. To confirm that DA and ACh systems were active simultaneously when the male's cloacal region contacted the vulva, we additionally measured Ca^+2^ transients in the male sex muscles: the gubernacular erector, the protractor and the anal depressor muscles (Figure S5). The contractile activities of these muscle cells are responsive to the ACh secretions of the PCB/PCC post-cloacal sensory neurons and the SPC motor neuron. In all of these muscles, Ca^+2^ transients increased when the male contacted the vulva (Figure S5) [24], [25]. Thus DA ray neurons likely down-modulate the simultaneously active cholinergic spicule protraction circuit during insertion attempts.

### Cholinergic spicule neuron stimulation causes Ca^+2^ transients in Rn7A

**Figure 4:**
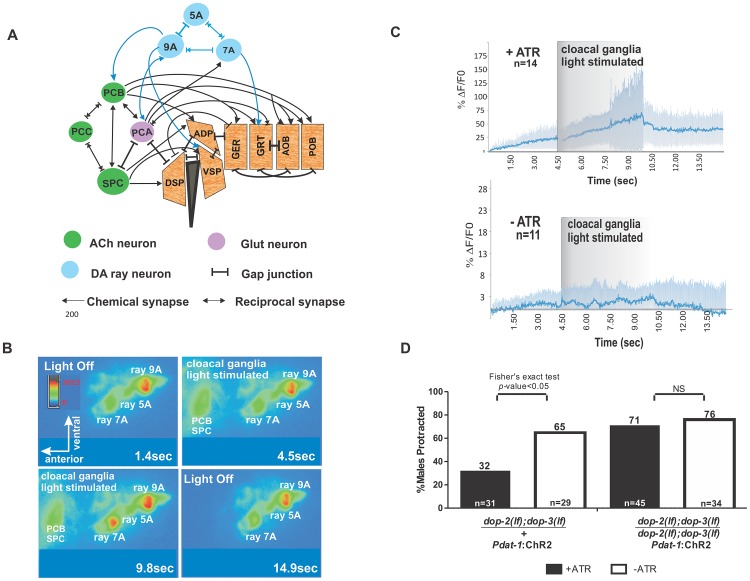
Activation of cloacal neurons increase Ca^+2^ transients in Rn7A, and activation of DA neurons attenuate ARE-induced muscle contraction via D2-like receptors. (A) Abridged schema of synapses between Rn5A, 7A, 9A and spicule circuit components. Arrows embedded in bars indicate reciprocal electrical and chemical synapses. Connections relevant to this work are depicted. For the complete wiring, refer to the male wiring project (S.W. Emmons, D.H. Hall, M. Xu, Y. Wang and T. Jerrel, Male Wiring Project, Albert Einstein College of Medicine, http://worms.aecom.yu.edu/pages/male_wiring_project.htm, [50]). Gubernacular erector (GER), gubernacular retractor (GRT), anterior oblique (AOB), posterior oblique (POB), dorsal spicule protractor (DSP), ventral spicule protractor (VSP) and anal depressor (ADP). (B) Video montage depicting changes in G-CaMP fluorescence in Rn7A. (C) The average %ΔF/F0 determined from all tested males used in both samples (top) with (n = 14) or (bottom) without (n = 11) ATR treatment representing Rn7A neuron Ca^+2^ transients before, during and after PCB, SPC stimulation. The dark and light blue lines represent the average and standard deviation values, respectively. (D) Males that protracted their spicules during simultaneous blue light stimulation of DA neurons and ARE exposure. The genotypes are written below each bar. The number of males tested and the percentage of spicule protracted males are listed at the bottom and top of the bars, respectively.

DA sensory ray neuron activity might be increased at the vulva because of direct vulval chemosensory stimulation, mechanical stimulation from pressing against the vulva or from humoral or synaptic stimulation from other cells. Since the increased activities of DA ray neurons and the spicule protraction neurons coincide during the insertion step of mating, we asked if ray neuronal activity could change as a direct or indirect response to PCB and SPC stimulation (Figure 4A). Therefore, we photo-stimulated PCB and SPC using channelrhodopsin-2 (ChR2), a light sensitive cation channel expressed from the *gar-3* mAChR promoter, while simultaneously recording DA ray neurons G-CaMP fluorescence (Figure 4B). The immobilized males were grown with or without all-*trans* retinol (ATR), a cofactor for ChR2. A microscope fitted with the mosaic imaging and illumination targeting system localized the blue light to the area of the G-CaMP-expressing ray neurons and then concurrently to the ChR2-expressing PCB, SPC neurons. We noticed that in ATR-grown males, the Ca^+2^ transients in Rn7A exclusively increased after PCB, SPC stimulation (n = 14 males) (Figure 4B and 4C, Figure S6, Video S2). However, no obvious dynamic Ca^+2^ changes were observed in Rn5A and Rn9A. These data suggest that Rn7A can respond, directly or indirectly to spicule circuit activity, whereas Rn5A and Rn9A likely respond to other signals.

Although the activity of the dopaminergic ray neurons is more dynamic when the male contacts the vulva, and D2-like receptors are expressed in the PCB neurons and sex-muscles (Figure S3), these ray neurons might attenuate the excitability of the spicule protraction circuits, not through endogenous DA and D2-like receptors, but through circuitous electrical signaling or other secreted neuropeptides. To address this, we photo-stimulated DA neurons by expressing ChR2 from the *Pdat-1* promoter, while simultaneously exposing the males to agar pads soaked with a concentration of ARE that causes ∼80% of males to protract their spicules within 5 minutes (Figure S1C). In a heterozygous *dop-2; dop-3/+; Pdat-1*:ChR2 background, 32% of the males protracted their spicules when photo-stimulated; however, in the homozygous *dop-2;dop-3(lf)* background, 71% of the photo-stimulated males protracted their spicules (Figure 4D). This suggests that endogenously evoked DA can attenuate ACh signaling in the spicule circuit via D2-like receptors.

### D2-like receptors promote spicule muscle contractile rhythmicity

**Figure 5:**
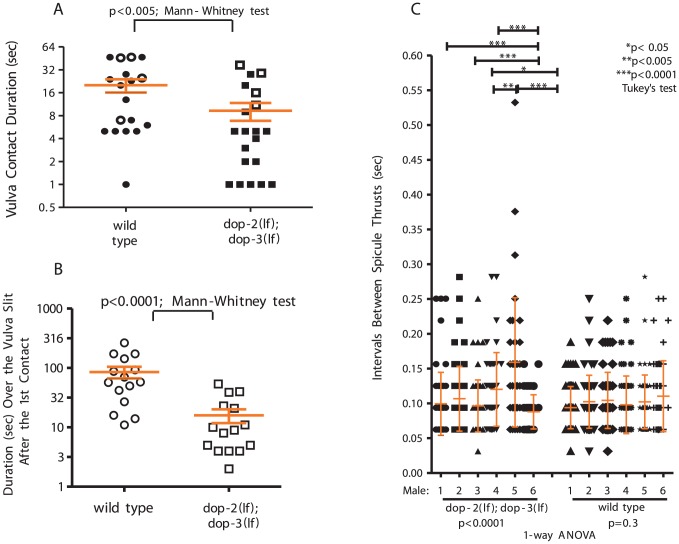
D2-like signaling promotes spicule insertion attempts. (A) Duration of vulval contact until spicule insertion or 120 sec. Symbols represent individual male performance. Open symbols represent unsuccessful insertions. (B) Duration over the vulval slit during the 1^st^ spicule insertion attempt. For A&B, line and error bars represent mean and SEM. (C) Spicule movement frequency calculated for 6 sec during spicule thrust against vulval slit. Symbols represent an individual frequency interval. Line and error bars represent mean and SD.

To ask how DOP-2 and DOP-3 regulate mating, we determined the mating potency of *dop-2; dop-3* mutant males with moving hermaphrodites. The mutant and wild type potencies were similar, 92% (n = 38) vs. 88% (n = 40), respectively. Thus, the functions of DOP-2 and DOP-3 are subtle. We then quantified *dop-2; dop-3* males' mating performance with paralyzed hermaphrodites and found that wild type, the double and single mutants behaved similarly during various mating steps (Figure S7). However, the double mutants can insert their spicules faster, into the paralyzed and easy-to-penetrate hermaphrodites, than wild type (Figure 5A). This paradoxical result suggests that having a wild-type version of D2-like receptors reduces reproductive fitness. However, males that lack D2-like receptors might not be at a behavioral advantage when paired with a more challenging mate. Therefore, we coupled *dop-2; dop-3* or a wild-type male with a moving hermaphrodite and directly measured the first vulval contact duration. We found that *dop-2; dop-3* males are displaced from the vulva faster than wild type (Figure 5B). Unlike wild type mating events, most hermaphrodites coupled with mutant males would abruptly move during spicule insertion attempts, causing the males to move off the vulva and thrust their spicules at areas adjacent to the vulva.

We previously showed that a K+ channel mutation disrupts the frequency and amplitude of sex muscle contractions during spicule insertion attempts; the arrhythmic spicule thrusts will startle the hermaphrodite and increase the probability of the male losing contact [29]. We reasoned that a similar phenomenon is occurring with *dop-2; dop-3* males. Thus, we measured spicule movement frequency when a male attempted insertion at a paralyzed hermaphrodite vulval slit. We found that relative to wild type, *dop-2;dop-3* spicule insertion attempts were less rhythmic. Among the assayed *dop-2; dop-3* males, the variability of durations between spicule thrusts was greater than compared to wild-type males, indicating that the mutants displayed more random sustained thrusts in runs of rapid shallow thrusts (Figure 5C, Figure S8).

### Restriction of non-productive mating behaviors requires D2-like signaling

**Figure 6:**
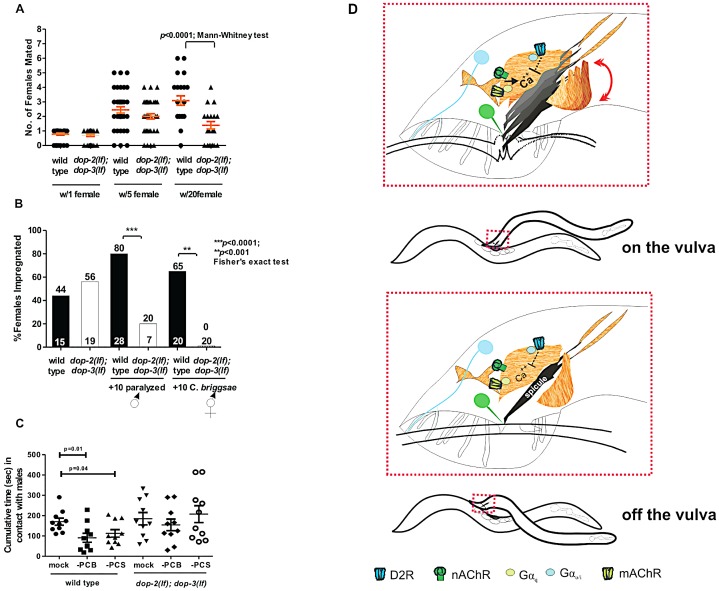
D2-like signaling promotes mating fitness. (A) Y-axis depicts the number of pregnant females amongst 1, 5 or 20 potential mates. Symbols represent individual male's sexual prowess. (B) Mating competition test pairing a wild type and a mutant male with one *fog-2* female +/−10 paralyzed males (first 4 columns), and number of *fog-2* females impregnated when wild type and *dop-2; dop-3* males were paired individually with 10 *C. briggsae* hermaphrodites ( last 2 columns). The top and bottom of each column indicates the % of pregnant females and the number of males that copulated or number of males assayed (last 2 columns). (C) The cumulative time in contact with males that a wild type and *dop-2; dop-3* male, represented by each symbol, spent when surrounded by 40–50 paralyzed males. Each data subset represents non-ablated animals (mock), PCB or PCS ablated animals. *p-values* calculated using the Mann-Whitney test. For A&C, line and error bars represent mean and SEM. (D) Top shows coincident D2-like and ACh signaling during spicule insertion attempts. Bottom depicts residual DA signaling when a male moves off the vulva. Blue and green color represents DA and ACh signaling components, respectively.

Since *dop-2; dop-3* and wild-type males behave differently during copulation with moving mates, we identified conditions where that difference would result in reduced mating fitness for the mutants. We paired either one wild type or one mutant male for 4 hrs with increasing numbers of moving *fog-2(lf)* virgin females (which contain a mutation disrupting self-sperm generation), and counted the number of impregnated females. Since mates with variable mating receptiveness exist in a population, the subtle defects of *dop-2; dop-3* males might be more evident in a competition to impregnate the most partners. Although wild type and mutant males' refractory periods between ejaculations are similar (Figure S9A), we found that when the female numbers increased, wild type impregnated more females than *dop-2; dop-3* males (Figure 6A). This difference was obvious when *dop-2; dop-3* males were exposed to 20 females. The lower serial mating potency is likely attributed to behavioral differences; however it could also be due to subtle germ line variations between the wild type and mutant males.

To address if behavioral differences caused the double mutants to impregnate fewer females, we simultaneously paired a mutant and a wild-type male with a single 1-day-old *fog-2* female and asked which male mated first. We found that *dop-2; dop-3* and wild type males impregnated a similar percentage of females (56% vs. 44%) (Figure 6B). Similar to Figure 6A data, this indicates that with a single mate, mutant males are similar to wild type in copulation. However, when we challenged the double mutant and a wild type male with a single *fog-2* female and 10 paralyzed males, as unproductive mating distractions, we found that wild type impregnated 80% of the females (Figure 6B). We observed that wild type and the mutant males contacted both sexes with equivalent frequency in this assay, and in a male-female mate choice assay, we did not find any indication that the mutant males had a greater chemotaxic preference to males (Figure S9B, S9C, and S9D). However wild-type males immediately terminated mating attempts with paralyzed males, whereas mutant males would abnormally scan and attempt spicule insertion into these inappropriate mates (Figure S9C). To rule out the possibility that *dop-2; dop-3* males displayed general locomotor hyper-exploratory behaviors, which could lead the mutant males to contact another animal before wild-type males, we compared the velocity and distance travelled of mutant and wild-type males during crawling. We found that there were no gross differences in these parameters between wild type and mutant males (Figure S10). Therefore, this indicates that during mating, D2-like signaling dampens ACh-induced behaviors with uncooperative/inappropriate mates.

Next, we addressed, in a more natural scenario, the importance of D2-like receptors in decreasing fruitless mating attempts with nematodes of other species. We paired one *fog-2* female and 10 *C. briggsae* hermaphrodites or 10 *C. remanei* females, with one wild type or a *dop-2; dop-3* male and counted how efficiently the *fog-2* female became impregnated. We found that after 4 hrs, wild type impregnated 65% more females than mutant males, when challenged with 10 *C. briggsae* hermaphrodites (Figure 6B and Figure S9E). In contrast, we found both wild type and mutant males' ability to impregnate a *C. elegans* mate is severely reduced when challenged with *C. remanei* females (Figure S9E), consistent with the published report that *C. remanei* females are more attractive than *C. elegans* hermaphrodites [51].This indicates that D2-like signaling might limit unproductive mating attempts with other hermaphroditic nematode species.

Finally we addressed whether D2-like signals specifically dampened spicule circuit excitability and/or other mating circuits, to restrict aberrant mating attempts. The male's response to contacting a mate is primarily facilitated by the ray sensilla. However, published reports have demonstrated that other male sensilla, such as the post-cloacal sensilla (p.c.s), spicule tips and possibly the hook sensillum can feebly substitute for ray function; therefore the activity of these sensilla might be increased in the *dop-2; dop-3* males [26], [27]. Since driving expression of DOP-2/DOP-3 exclusively in cells of the spicule circuit is not technically possible, we opted for an alternative approach of laser ablating the *dop-2*-expressing PCB neuron or all of the p.c.s neurons (PCA, PCB and PCC), and asking if mating with a non-hermaphrodite is reduced. First we quantified in wild-type males lacking PCB or all 3 p.c.s. neurons, the cumulative and average duration in contact with another male during a 10 min assay period, when surrounded by 40–50 paralyzed males. We found that the cumulative time that operated males spent with other males was reduced (Figure 6C). This result is consistent with the idea that the post-cloacal sensilla play a minor role in contact response and scanning behavior. However if an operated male does initiate scanning with another male, the average time was not significantly different amongst these groups (Figure S9F). We then tested if PCB or p.c.s ablations in *dop-2; dop-3* males would reduce abnormal mating attempts. We found that *dop-2; dop-3* males on average spent longer amount of times scanning other males than wild type (Figure S9F); however, neither PCB nor p.c.s ablations reduced this phenotype. In addition, the cumulative time in contact with another male was similar between operated and intact males (Figure 6C). This indicates that D2-like signaling must be modulating other circuits in addition to the post-cloacal sensilla to attenuate contact response and scanning behavior.

## Discussion

Although dopamine (DA) modulation in vertebrate models is known to regulate motor patterns [2], [7], there are few in-depth analysis for how DA fine-tunes context-dependent behaviors. To address this, we analyzed how DA signaling constrains specific neuromuscular outputs during *C. elegans* mating. As the male positions his tail over the vulva, he repetitively thrusts his spicules against the vulval slit while adopting an arched posture. This behavior is terminated after spicule penetration or loss of vulval contact. In contrast, in DA-deficient *cat-2* males arched postures and rhythmic spicule insertion attempts were no longer confined to the vulval region, and sometimes even initiated randomly on the hermaphrodite. Thus, spicule motor behaviors coupled with appropriate postures and vulva sensing are partially coordinated by DA down-modulatory pathways.

Consistent with the *cat-2* male's ectopic display of motor behaviors, simultaneous application of exogenous DA with receptor-selective or nonselective acetylcholine (ACh) agonists constrains cholinergic-mediated sex-muscle contraction. Interestingly, the inhibitory effect of exogenous DA is less potent with a muscarinic (G-protein coupled receptor) agonist or a muscarinic and ionotropic (ACh-gated ion channel) nonselective ACh agonist, if DA is applied first. This suggests that during mating, context-relevant DA signaling occur coincidently with ACh-mediated signaling; additionally, mAChR signaling might make the spicule circuit refractory to non-coincident humoral DA secretions that occur elsewhere in the male.

DA-dependent negative signals are partly transduced through the D2-like G-protein-coupled receptors DOP-2 and DOP-3. Even though *dop-2*; *dop-3* double mutant phenotypes are less severe than *cat-2* animals, likely because every DA receptor is affected by the *cat-2* mutation, we found that these receptors mediate restriction of spicule protraction behavior to the precise vulval slit area, and maintain rhythmic spicule thrusts during penetration attempts. Although previous reports demonstrate DOP-3 and GOA-1 signaling for hermaphrodite locomotion [21], [23] and *in vitro* DOP-2 and GPA-14 interactions [52], we provide genetic evidence that Gα_o/i_ proteins, GOA-1 and GPA-7, redundantly transduce DA inhibitory signals during vulva sensing/spicule insertion behavior (Figure 6C). These Gα_o/i_ proteins, and their βγ partners might regulate molecules such as adenylyl cyclase, L-type- voltage-gated Ca^++^ channels and K^+^ channels to decrease neuromuscular excitability [53]–[55].

ACh secreted from the cloacal ganglia sustains vulval contact [24], while concurrently, the 9 pairs of sensory rays likely provide feedback to adjust the male's movement and posture according to the hermaphrodite's position and locomotion. Three of the 9 ray pairs contain DA sensory neurons; when optogenetically stimulated, they induce a shallow ventral tail flexure [26], and when stimulated in the presence of non-selective ACh agonists endogenous DA secretion antagonizes spicule protraction. The 3 pairs of RnA neurons gap junction to their RnB counterparts, which express neuropeptides *flp-5, flp,-6*, and *flp -17* [*56*], raising the possibility that stimulation of RnA neurons indirectly leads to neuropeptide-dependent modulation of the spicule circuit. However in the *dop-2; dop-3* double mutants, ChR2-stimulation of *dat-1* expressing cells, and possibly including the RnB neurons via gap junctions, failed to reduce simultaneous ACh agonist-evoked contractions. This suggests that DA secretions, possibly from Rn5A, Rn7A and Rn9A, can attenuate the output of cholinergic signaling. Of these neurons, Rn7A and 9A make chemical synapses to cloacal-associated components (Figure 4A), suggesting that DA-ACh signals might be involved during the vulva location/spicule insertion steps.

In fact, the DA ray neurons and the spicule circuit components are more dynamic during vulval contact. However, heightened ray neuronal activity is sometimes detected when the male is at non-vulval regions, suggesting that DA secretion is not tightly coupled to an explicit sensory signal. Our optogenetic experiments indicate that cholinergic cloacal neuron activation stimulated Rn7A activity This DA-secreting cell is not post synaptic to the cholinergic cloacal neurons, indicating that DA secretion might be an indirect response to cholinergic circuit activity via humoral signaling or interneurons. Additionally, the Rn5A and Rn9A did not responded to PCB and SPC stimulation, further suggesting implications of additional internal signals from the locomotor circuit or other ray neurons.

Throughout mating, ray neurons respond with an array of different dynamics correlated with the gradual arched body posture changes, which are perhaps a read-out of DA not only modulating the PCS, but also providing feedback onto a locomotor circuit. This is a possibility since DOP-2 and DOP-3 are expressed in ventral cord neurons and body wall muscles [21], which facilitate locomotion. During non-arched postures, either scanning or at the vulva, DA ray neurons display stable activity. However; if a male develops an arched posture at the vulva, or during scanning, then the DA ray neurons display dynamic changes in their activity, maybe to modulate the transition in overall motor response. During male behaviors, we have observed a similar spicule circuit independent modulatory role for DA and D2- receptors, where reduction in D2-like signaling results in mutants engaging in prolonged backward scanning locomotion with other males and hermaphrodites of different species. Therefore, the DA-signaling mutant phenotypes, together with the expression of DA ray neuronal activity, suggest that DA refines motor outputs at the vulva and delimits them at other areas via interactions with neural-muscular networks that include the spicule protraction circuit. This ACh/DA interplay might share analogy with how the vertebrate CNS fine-tunes locomotor control.

In the vertebrate CNS, DA secretions from the substantia nigra (SN) inhibit striatal ACh interneurons, and ACh-induced DA release in these networks coordinate voluntary movements [10], [57]. Although this suggests bidirectional DA/ACh signaling in the CNS, direct evidence for how these neurons shape motor outputs at the animal behavioral level is scarce, due to complex CNS connectivities [54], [58]. In the *C. elegans* male, the DA ray neurons and cholinergic cloacal ganglia interact bi-directionally to regulate sex-muscle behaviors. The optogenetic experiments suggest that cloacal ganglia neurons promote DA- ray activity and the pharmaco-genetic experiments indicate that DA attenuates the ACh spicule circuit output partly via DOP-2 and DOP-3 on PCB neurons and sex muscles. Decrease of PCB output could subsequently result in reduced DA secretion and dampening of DA and ACh circuits' interactions.

During mating, how can a male insert his spicules while attenuating DA signaling occurs? Possibly during the repetitive vulval penetration attempts, potent ACh secretions can override DA-negative signaling, due to acute vulval stimulation of the cloacal sensory-motor neurons. However, these cloacal neurons make reciprocal (recurrent) synapses with one another (Figure 4A). If the wild-type male moves off the vulva or if these neurons are inappropriately stimulated (on non-vulval regions, on another male, or by a mate from a different species), then a negative mechanism, such as D2-like signaling, must dampen the circuit's residual self-amplifying property. Indeed, the ectopic mating behaviors displayed by *cat-2* and *dop-2; dop-3* males give the illusion that they compulsively maintain motor behaviors (spicule prodding ) in the absence or withdrawal of the appropriate stimuli. The ACh and D2-like signaling interactions in *C. elegans* are reminiscent of D2 receptor-regulated synaptic plasticity in vertebrate SN-striatal networks. In these networks, D2 receptors regulate long term synaptic depression. This form of plasticity reduces pre-existing motor memory storage and maintains a balance between old and newly encoded motor information. In DA-deficient Parkinson's disease models, dyskenisia (voluntary movement disorder) is caused by plasticity abolishment in these networks [12], [59]–[62].

## Materials and Methods

### Strains and culture methods

Strains were maintained at 20°C on NGM agar plates and fed with *E. coli* OP50. All *C. elegans* males contain the allele *him-5(e1490)*. Additional alleles used were: *cat-2(e1112), dop-1(vs100), dop-2(vs105), dop-3(vs106), dop-4(tm1392), goa-1(n363), gpa-7(pk610), gpa-14(pk347), gpa-16(it143), pha-1 (e2123), unc-64(e246), fog-2(q71), unc-29(e193), gar-3(gk305)* and *acr-16(ok789)*.

Transgenic strains include: *pha-1(lf); lite-1(lf); rg*Ex197[ P*unc-103E*:*G-CaMP1.3*, P*unc-103E*:*mDsRed*, *pha-1*(+)], *pha-1(lf); lite-1(lf); rg*Ex317[P*dop-2*:*ChR2*::*YFP*; *pha-1*(+)], *pha-1(lf); lite-1(lf); rg*Ex326[P*tph-1*:*CFP*; *pha-1*(+)], *pha-1(lf)*; *lite-1(lf)*; *rg*Ex431[P*hsp-16*:*egl-2(n693gf)*cDNA; P*unc-103E*:mDsRed; *pha-1(+)*], *dop-2(lf); pha-1(lf)*; *lite-1(lf) rg*Ex462 [P*aex-3*:*dop-2*::*CFP*], *dop-2; pha-1(lf)*; *lite-1(lf); rg*EX467 [P*unc103E*:*dop-2*::*CFP*], *dop-3; pha-1(lf); rg*Ex482 [P*unc103E*:*dop-3*::*YFP*]; *dop-3; pha-1(lf); rg*Ex490 [P*aex3*:*dop-3*::*YFP*], *pha-1(lf); lite-1(lf); rg*Ex491[P*gpa-7*:*YFP*; *pha-1*(+)], *pha-1(lf); lite-1(lf); rg*Ex512[P*gar-3*B:*GCaMP3*::*SL2*:::*mDsRed*; *pha-1*(+)], *dop-2(lf); dop-3(lf); rg*Ex515[P*tph-1*:*YFP*], *pha-1(lf); lite-1(lf); rg*Ex517[P*dat-1*:*GCaMP3*::*SL2*:::*mDsRed*; *pha-1*], *pha-1(lf); lite-1(lf); rg*Ex519[P*gpa-16*: *gpa-16* exon1::*YFP*; *pha-1*(+)], *pha-1(lf); lite-1(lf); rg*Ex523[P*dat-1*:*G-CaMP3*::SL2:::*mDsRed*, P*gar-3*B:*ChR2*::*YFP*, *pha-1(+)*], *pha-1(lf); lite-1(lf); rg*Ex549[P*dat-1*:*G-CaMP3*::SL2:::*mDsRed*, P*dat-1:unc-103(gf)*, *pha-1(+)*], *dop-2(lf); dop-3(lf); pha-1(lf); rg*Ex550[P*dat-1*:*ChR2*::*YFP*, *pha-1(+)*]

### Plasmids

#### Reporters of *dop-2*, *gpa-7*, *and gpa-16* expression

Primer sequences are provided in Table S2. A 9.2 kb region upstream of the *dop-2* ATG was PCR-amplified using the primers: ATTB1Dop2pr and ATTB2Dop2pr. A 3.1 kb region upstream of the *gpa-7* ATG and the first four codons was PCR-amplified with the primers: ATTB1gpa-7F and ATTB2gpa-7R. A 2.6 kb region upstream of the *gpa-16* ATG, exon1 and 34 codons of exon2 was PCR-amplified with the primers: Pgpa-16Fv2 and Pgpa-16Rv2. These primers contained Gateway ATTB sites, which allowed the *dop-2, gpa-7*and *gpa-16* PCR products to be recombined using BP clonase (Invitrogen, CA), into the low copy number Gateway entry vector pDG15 [63], to generate pPC1, pPC24 and pPC40, respectively. To place the dopamine receptor and G-protein sequences upstream of YFP, these vectors were recombined with YFP destination vectors. pPC1 was recombined with pLR167 (a plasmid containing the gateway destination AttR Reading frame Cassette C.1 upstream of the channel rhodopsin fusion protein ChR2:YFP) [24]; pPC24 and pPC40 were individually recombined with pGW322YFP (a low-copy plasmid containing the gateway destination AttR Reading frame Cassette C.1 upstream of YFP) [63] using LR clonase (Invitrogen) to make plasmids pPC2, pPC39, pPC41, respectively.

#### Cell-specific expression of *dop-2* and *dop-3* genomic DNA

A 5.2 kb genomic *dop*-2-containing sequence from the ATG to last valine codon was PCR-amplified via primers Attb1DOP2F and Attb1DOP2R (Table S2). Since these PCR primers contained Gateway ATTB sites, *dop-2*(genomic DNA) was recombined using BP clonase, into pDG15, to generate pPC9. To make pPC11 [*dop-2::CFP*], pPC9 was recombined with pGW77C (a high-copy plasmid containing the gateway destination AttR cassette upstream of CFP [32] using LR clonase. The LR sites flanking DOP-2 were removed using single site mutagenesis to obtain the pPC15 plasmid. To drive *dop-2::CFP* expression from different promoters, pPC15 was cut with *Afe*III and Gateway Vector Conversion Reading frame Cassette B(Invitrogen) ligation generated the destination vector pPC16. To drive *dop-2::CFP* expression from the *dop-2* endogenous promoter (*Pdop-2*), a sex-muscle expressing promoter (P*unc-103E*) and a pan-neuronal promoter (P*aex-3*), the plasmids pPC1, pLR21 [63] and pLR35 [33] were individually recombined into pPC16 using LR clonase, to make pPC21 [P*dop-2* :*dop-2*::*CFP*] and pPC18 [P*aex-3*:*dop-2*::*CFP*] and pPC19 [*Punc103E*:*dop-2*::*CFP*], respectively. A 5.2 kb genomic *dop*-3-containing sequence from the ATG to last cysteine codon was PCR-amplified via primers DOP3geneF and DOP3geneR (Table S2). To fuse DOP-3 C-terminal end to YFP, the PCR product was cut with *BamH*I and *Age*I, and then cloned into the YFP-containing plasmid pSX322 *BamHI* site [63] to generate pPC23. To drive *dop-3::YFP* expression, pPC23 was cut with *BamH*I and ligated with the Gateway Vector Conversion Reading frame Cassette C.1(Invitrogen) to generate the destination vector pPC33. To make sex-muscle specific and pan-neuronal *dop-3::YFP* expression vectors, the plasmids pLR21 and pLR35 containing *Punc-103E* and *Paex-3*, respectively, were recombined into pPC23 using LR clonase, to generate pPC36 [P*unc-103E* :*dop-3*::*YFP*] and pPC37 [P*aex-3*:*dop-3*::*YFP*].

#### G-CaMP3 plasmids

We inserted an SL2-accepting *trans*-splice site followed by the mDsRed gene and an *unc-54* 3′UTR immediately downstream of Gateway AttR Reading frame Cassette C.1and G-CaMP3 [64] to create the vector pLR279. To introduce promoters upstream of the G-CaMP and DsRed sequences, the plasmids containing the promoters: P*gar-3B*(pLR57) [28] and P*dat-1*(pZL15) [26] were recombined with pLR279 to generate the plasmids pLR283 and pLR286,respectively.

#### Plasmids used for hyper-polarization and stimulation of DA neurons

We introduced the *Pdat-1* upstream of the *unc-103(gf)* and ChR2 sequences to hyper-polarized and stimulate DA neurons respectively. The plasmid containing the *dat-1* promoter (pZL15) was recombined with pLR279 and pLR167 [31]to generate the plasmids pPC47 and pPC48.

### Transgenics

Plasmids were co-injected with pBX1(50 ng/µl) [65] into strains that contained the *pha-1(e2123)* allele. Transgenic lines that could stably propagate at 20°C were kept for further analysis. For strains that did not have the *pha-1* allele, CFP or YFP expressed from one of the injected plasmids was used to identify transgenic animals. For all injections, pUC18 was used to make the final DNA concentration 200 ng/µl. The expression constructs pPC2 and pPC39 were injected at 100 ng/µL into *pha-1 him-5 lite-1* hermaphrodites. To rescue the *dop-2(lf)* and *dop-3(lf)* DA+ARE sensitivity, pPC21, pPC19, pPC18 (25 ng/µL) and pPC36, pPC37 (50 ng/µL) were injected into *dop-2; pha-1* and *dop-3; pha-1* hermaphrodites, respectively. To fluorescently label males for mating competition tests, pTG10 [*Ptph-1*:CFP](100 ng/µL) and pTG11[*Ptph-1*:YFP ](100 ng/µL) [30] were injected into *him-5* and *dop-2(lf); dop-3(lf)* hermaphrodites, respectively. The *goa-1(lf); gpa-7(lf)* strain was injected with pPC41 [*Pgpa-16*:*gpa-16*::*YFP*] (100 ng/µL) to test for RNAi effectiveness. To label separately the dopamine-expressing cells, the male cloacal neurons and the male sex muscles with G-CaMP3::SL2:::mDsRed, *pha-1; him-5*; *lite-1* hermaphrodites were injected with pLR286[P*dat-1*:G-CaMP3::SL2:::mDsRed](30 ng/µl), pLR283[P*gar-3*B:G-CaMP3::SL2:::mDsRed] (30 ng/µl) or pLR289[*Punc-103*E:G-CaMP3::SL2:::mDsRed](30 ng/µl), respectively. To co-express G-CaMP3 in dopamine-expressing cells and Channel Rhodopsin in the male cloacal cells, *pha-1; him-5; lite-1* hermaphrodites were injected with a mixture of pLR286[P*dat-1*:G-CaMP3::SL2:::mDsRed](30 ng/µl), and pLR183[P*gar-3*B:ChR2::YFP](100 ng/µl) [24]. To co-express G-CaMP3 and *unc-103(gf)* in dopamine-expressing cells, *pha-1; him-5; lite-1* hermaphrodites were injected with a mixture of pLR286[P*dat-1*:G-CaMP3::SL2:::mDsRed](30 ng/µl), and pPC47[P*dat-1:unc-103 (gf)*](70 ng/µl). The *dop-2; dop-3; pha-1* strain was injected with pPC48 [P*dat-1*:ChR2::YFP](70 ng/µl) and then crossed into *pha-1; him-5; lite-1* to obtained the heterozygous strain carrying the same transgene for optogenetic experiments. To obtain a strain for behavioral comparisons with transgenic males [P*dat-1*:G-CaMP3::SL2:::mDsRed], the *pha-1; him-5; lite-1* hermaphrodites were injected with pPC46 [P*dat-1*:YFP].

### Behavioral assays and drug test

For behavioral and pharmacology assays, virgin males were isolated from non-crowded plates, either at the late L4 stage (when cells in the male tail spike have completely migrated anteriorly) or after they newly crawled out of their L4 cuticle. They were kept solitary or in groups of 10–20. All drug tests scored the number of males that protracted their spicules by directly observing spicule protraction for at least 10 seconds in a 5 min observation window; these behavioral assays were not videotaped. If multiple mating parameters were measured for individual males, we videotaped the mating event from the time a male contacted a hermaphrodite until spicule insertion. Because all of the sensory-motor metrics were objective (% fluorescent changes, motor duration, contact duration, number of contacts, locomoter velocity, sex muscle contraction frequency), and not subjectively defined, it was not necessary to collect data blinded to the genotype of the animals. For populations of objective metrics that were statistically different, but less than twofold between the experimental and control animals, two observers, Paola Correa and L. Rene Garcia, re-analyzed the movies independently to re-verify or amend the results. Graphpad Prism 5 software was used to perform all statistics. Fisher's exact test was used when comparing categorical variables (protracted vs. non-protracted, potent vs. non-potent). The Mann-Whitney nonparametric test was used to compare the metrics of an experimental group with a control group, when the data did not fall under a normal Gaussian distribution. When the data fitted a Gaussian distribution, 1-way ANOVA and Tukey's post-test were used to compare the means and standard deviations of more than two groups.

To assay agonist-induced spicule protraction, we dissolved levamisole (LEV) (ICN Biomedicals, OH), arecoline (ARE) (Acrose organics, NJ) , nicotine (NIC) (EM, NJ), oxotremorine M (OXOM) (Sigma, MO) and dopamine (DA) (Sigma) in water to make a stock solution of 10 mM, 10 mM, 100 mM , 50 mM and 30 mM, respectively. We added between 400–1000 µL of the drug to a three well round-bottom Pyrex titer dish. Five to ten males were then transferred to the drug bath and observed for five minutes at 20°C. Males were considered drug responsive if their spicules remained protracted for ≥5 sec. For simultaneous exposure, DA and ACh-agonists were pre-mixed. For sequential exposure, worms were bathed in DA for 1 min and then ACh-agonists were added at a concentration, such that the final DA concentration was not significantly changed and the ACh-agonists were at the EC90 concentration.

For mating potency tests, 10 µl of a saturated *E. coli* culture was spotted onto a NGM agar plate, to make a 3.5 mm lawn. ∼20 hr later, a single male and a single adult virgin *pha-1(lf)* hermaphrodite were put onto the mating lawn and incubated at 20°C for 4 days. A male was considered potent if the plate contained cross-progeny. For mating behavioral assays, we spaced ten 48 hr-old *unc-64(lf)* adult hermaphrodites on a 5 mm diameter bacterial lawn and placed a male in the lawn's center. Movies were recorded using a stereomicroscope mounted with a Hamamatsu ImagEM CCD camera (Hamamatsu, USA); recordings were taken from the time a male contacted a hermaphrodite until spicule insertion or 5 min. Different mating performances were scored from observations of these recordings to address: the number of times a male contacted the vulva, total duration of vulval contact and the time a male spent scanning a hermaphrodite. The same population of males was used to obtain these data sets for each behavioral metric. Wild type and mutant males were tested in parallel for statistical comparisons. Through direct observation and using a hand-held timer, we measured the time it took wild type and *cat-2(lf)* males to contact and start scanning a hermaphrodite. Moving hermaphrodites were used to measure the duration over the vulval slit after the 1^st^ contact for wild type and *dop-2(lf); dop-3(lf)* males.

To determine if *dop-2; dop-3(lf)* males differ from wild type in their chemotactic or locomoter behaviors toward paralyzed *pha-1*; *lite-1;him-5; rg*Ex431[P*hsp-16*:*egl-2(n693gf)*cDNA; P*unc-103E*:mDsRed; *pha-1(+)*] hermaphrodites or males, 6 paralyzed hermaphrodites and 6 paralyzed males were alternately and equally positioned at the periphery of a 1.5 cm diameter OP50 lawn. One wild type or *dop-2; dop-3(lf)* male was placed at the center of the lawn and allowed to crawl around for up to 5 minutes. The males were timed when they first placed the ventral side of their tail against the cuticle of a paralyzed worm (either male or hermaphrodite) for greater than 1 second.

To determine the male's movement velocity, an 18–24 hr adult virgin male was placed in the center of a thin 3 mm OP50 lawn. The forward crawling animal was digitally recorded for 1 minute at 30 frames per second using a stereomicroscope mounted with a Hamamatsu ImagEM CCD camera (Hamamatsu, USA). The lighting of the sample was adjusted to maximize the contrast of the male against the bacterial lawn. Recordings were then analyzed using the Hamamatsu SimplePCI (version 6.6.0.0) software to determine the centroid of the male in each frame, and track the changes in the X and Y coordinates of the centroid as the male crawls forward. Microsoft Excel was then used to convert changes in the X and Y coordinates into the velocity and distance traveled during the 1 minute recordings.

For mating assays with multiple mates, a one-day-old wild type or *dop-2; dop-3(lf)* male was paired with 1, 5, or 20 two-day-old *fog-2(lf)* females in a plate containing a small bacterial lawn. After 4 hrs, the male was removed. The number of females that laid eggs were determined 4 hours later and then subsequently monitored for an additional 18 hrs. In experiments where wild type or *dop-2; dop-3* males must discriminate between *C. elegans* and either *C. remanei* or *C. briggsae*, L4 *fog-2(lf)* females were grown to adulthood on OP50-seeded NGM agar plates containing 50 µM red fluorescent dye SYTO-17 (Invitrogen, Eugene OR); the dye allowed the *fog-2(lf)* females to be identified from *C. remanei* or *C. briggsae* animals. One stained virgin 18–24 hrs adult *fog-2(lf)* female was placed with ten 18–24 hrs adult *C. briggsae* hermaphrodites or 10 virgin *C. remanei* females on a 3.5 mm diameter lawn of OP50. The animals were allowed to acclimate to the lawn for one to two hours before a single virgin wild type or *dop-2; dop-3* male was introduced. Males were kept continuously with their mates for 18 hrs. Four and 18 hours later after the male was first introduce with his mates, using an epi-fluorescence-equipped stereomicroscope, we determined if SYTO-17 stained *fog-2(lf)* females contained eggs in the uterus or sperm in the spermatheca.

To observed how post-cloacal sensilla-ablated wild type and *dop-2; dop-3(lf)* males behave with paralyzed males, the cells PCA, PCB and PCC were laser ablated (using a Spectra-Physics VSL-337ND-S nitrogen laser attached to an Olympus BX51 microscope via the MicroPoint laser focusing system) in L4 males prior to tail spike retraction. During the operation, the laser-ablated and mock-ablated males were immobilized between a microscope coverslip and an 8% noble agar pad (a higher % pad caused the males to rupture through their anus) containing Polybead polystyrene 0.1 µm microspheres (Polysciences, Inc., WA). Eighteen to 24 hrs later, 3–4 laser-ablated or mock-ablated adult males were added to a 3 mm diameter OP50 lawn that contained 40–50 paralyzed *pha-1*; *lite-1; him-5; rg*Ex431[P*hsp-16*:*egl-2(n693gf)*cDNA; P*unc-103E*:mDsRed; *pha-1(+)*] males. The animals were digitally recorded at 1 frame per second for 10 minutes. The recordings were then reviewed, and time and duration that the moving male placed the ventral side of his tail against the cuticle of a paralyzed worm or another moving worm for greater than 1 second was determined for the whole 10 min recording. Cumulative time was calculated by adding up the total time a male was in ventral contact with other males. The average time was calculated by dividing the total time in contact by the number of mating contacts.

Because the mating behavioral steps that lead up to sperm transfer can be highly variable, we required a metric to score/rank a spectrum of behavioral responses that result in successful spicule insertion. We wanted that metric to differentiate a male that instantly found the vulva and inserted within a second or two of contact, from a male that meandered around the hermaphrodite for 110 secs, but eventually contacts the vulva, and inserts. However, this metric must be able to rank the spectrum of males that immediately undergo spicule insertion attempts and are persistent, but are unsuccessful in penetration, with males that were erratic in prodding behavior (and other steps of mating behavior), but fully inserted their spicules. To achieve this, the metric had to measure the period between vulval contact and full insertion, but it also had to incorporate a penalty for not being diligent at immediately initiating vulval spicule insertion behavior after contacting with a mate, and a bonus if successful penetration occurred, even after erratic performance of other mating behavioral steps. The efficiency of spicule insertion, E_SI_, was calculated from recordings made during the first 120 seconds of contact between the male and a paralyzed 2-day-old hermaphrodite. The metrics recorded were: (1) duration of prodding at the vulva; (2) duration in contact with the hermaphrodite at areas outside the vulva. If the male successfully inserted his spicules before the 120 seconds were over, then the observation was stopped. E_SI_ = (time (sec) spent at spicule insertion attempts/total time (sec) in contact with hermaphrodite, up to 120 sec) X (1/time (sec) in contact with the hermaphrodite, such scanning, but not attempting insertion (penalty)) X (1+ (0 if no penetration, otherwise time (sec) remaining after a successful penetration, /120 sec)(bonus)). A hypothetical E_SI_ of 1.99 would mean that the male located the vulva and inserted his spicules approximately 1 sec after contact with the hermaphrodite; whereas a hypothetical E_SI_ of 0.0 meant that the males spent their first 120 seconds of contact not attempting spicule insertion at the vulva [66].

For mating competition tests, transgenic males contained expressed YFP or CFP from the *tph-1* promoter [30]. Mid to late L4 *fog-2* females were separated from males; 48 hrs later, a single female was added to 5 mm diameter lawns of bacteria. One 18–20 hrs virgin CFP-expressing wild type and one YFP-expressing *dop-2(lf);dop-3(lf)* male were added simultaneously to the lawn. The plates were incubated at 20°C for one hour. If the female had sperm in the spermatheca (determined via standard bright field microscopy), then both males were removed, otherwise the animals were allowed to mate for another hour. Majority of the females were mated within 1 hour; by 2 or 3 hours, all females were impregnated. The next day, the fluorescence color of serotonergic neurons in the L1 cross-progeny was determined.

For the mating competition test with paralyzed males, *rg*Ex431 males containing a heat shock promoter-driven *egl-2(gf)* construct were incubated for 30 min at 30°C. After 3 hrs, the heat shocked-expressed EGL-2(gf) K+ channels caused complete paralysis. Ten paralyzed males were placed onto a mating lawn with a single *fog-2(lf)* female. One CFP-expressing wild type and one YFP-expressing mutant male were simultaneously placed in the middle of the plate. The first male to insert was determined via observations and subsequently identified using fluorescent microscopy.

For mating assays with multiple hermaphrodites, a one-day-old wild-type or a *dop-2; dop-3(lf)* male was paired with 1, 5, or 20 two-day–old *fog-2(lf)* females in a plate containing a small bacterial lawn. After 4 hrs, the male was removed. The number of egg-gravid females were determined 4 hours later and then subsequently monitored for an additional 18 hrs.

### Ca^2+^ imaging and optogenetics

The genetically encoded Ca^2+^ indicator G-CaMP1.3 was used to visualize calcium transients in the sex muscles, and G-CaMP3 was used to visualize Ca^2+^ transients in neurons. A 2 cm square chunk from an NGM plate containing a 3 mm diameter OP50 lawn was placed on a microscope slide. 10–15 heat shocked paralyzed *pha-1; lite-1; him-5; rg*Ex431[P*hsp-16*:*egl-2(n693gf)*cDNA; P*unc-103E*:mDsRed; *pha-1(+)*] hermaphrodites were then evenly spaced on the lawn. The hermaphrodites were allowed to condition the lawn for ∼20 min before a male was added. One 18–20 hrs adult virgin transgenic male was placed on the lawn without a microscope coverslip and immediately placed on an epifluorescence–equipped Olympus BX51 microscope (Olympus, USA). Matings were visualized using a 10×, or 20× long working distance objective. Males were not exposed to high intensity filtered blue and green light until they initiated mating behavior. Exposure to the high intensity blue light, even though the males contain the *lite-1* mutation, interferes with the contact response step of mating. As the males were being recorded, the stage position and focusing were actively manipulated to keep the fluorescent cells in focus and in the center of the viewing field. New mating lawns were used after every two observations; long exposures to high intensity light affect the *E.coli* lawn in an unknown way that reduces the males' mating response.

The G-CaMP and DsRed fluorescence signals at the male tail were recorded simultaneously using a Dual View Simultaneous Image splitter (Photometrics, AZ) and a Hamamatsu ImagEM Electron multiplier (EM) CCD camera, at the speed of ∼30 frames per second. The Ca^2+^ data was analyzed using the Hamamatsu SimplePCI (version 6.6.0.0) software and Microsoft Excel, as described previously [24], [30].

The recordings were reviewed to find the first instance of an uninterrupted behavioral step (either moving forwards or backwards along the hermaphrodite cuticle, or attempting spicule insertion at the vulva) with a duration of 6 seconds or greater. Region-of-interests (ROIs), of equal areas, were generated in the Simple PCI software. The individual ray 5,7,9A neurons were too close to one another to separate with different ROIs, and thus their composite fluorescence was measured with a single ROI. The male gubernacular erector muscle, anal depressor and ventral protractor muscles were far enough so that separate ROIs could be drawn for each muscle. ROIs were used to measure the background and cellular fluorescence signals in both the green and red emission channels. The positions of the ROIs were manually adjusted for every frame in the movies. The mean pixel intensity (MPI) was measured for every ROI in every frame, in each recording (Figure S11B and S11C). The data was then transferred from Simple PCI to Microsoft Excel. For each recording frame, background ROIs values were then subtracted from their respective ROIs that quantified neuronal or muscular fluorescence (Figure S11D).

Focusing/gross movement/muscle contraction/mercury arc lamp flicking and photobleaching artifacts caused non-interesting fluorescence changes in both channels and in every frame. In some cases, a higher rate of mDsRed photobleaching, relative to the minimal G-CaMP photobleaching, made a simple green-to-red fluorescence ratio-metric analysis not appropriate to use. To correct this, the red channel was used as a reference to analyze the green channel. In each frame, the red channel background-subtracted MPI for each ROI was plotted with respect to time, and an average line (Figure S11E) or a one-phase decay curve (to correct for mDsRed specific photobleaching) was fitted over the data points using GraphPad Prism (version 4.03). The fitted curve serves as an arbitrary reference to quantify the magnitude of non-interesting fluorescence changes that occurred in each frame. For each frame, the measured background subtracted red channel MPI value was divided by the average or fitted red value to give a correction value. The corrected inverse value for each frame was then multiplied to the subtracted green channel MPI of the respective frame (Figure S11F). This corrects the values for the green channel, so that the fluorescence changes reflect calcium transients rather than gross experimental artifacts. The values for each recorded frame was then calculated as ΔF/F0 = (((corrected MPI (frame n)-corrected MPI (frame 0(initial frame)))/corrected MPI (frame 0(initial frame))) ×100) (Figure S11G). The arbitrary F0 value was determined as the fluorescence value in the first frame of the recordings. The values were then plotted with respect to time.

To determine whether the G-CaMP3 transgene might severely interfere with the behaviors displayed by the males, we quantified the mating behavior of the *Pdat-1:G-CaMP3* strain. In a mating potency test, these males sire progeny similar to wild type (Figure 1A). During mating, the vulval contact duration, number of vulval contacts and time in contact between insertion attempts in these males were also similar to males carrying the *Pdat-1:YFP* transgene (Figure S2D–S2F). This indicates that any observed changes occurring in ray neurons of the *Pdat-1:*G-CaMP3 strain portray a biological relevant phenomenon possibly occurring during wild type mating.

For the optogenetic analyses, *rg*Ex523[P*dat-1*:*G-CaMP3*::*SL2*:::*mDsRed*, P*gar-3*B:*ChR2*::*YFP*, *pha-1*(+)] males, incubated +/− with all-*trans*-retinal, were immobilized between a microscope coverslip and a 10% noble agar pad containing Polybead polystyrene 0.1 µm microspheres (Polysciences, Inc., WA). In the cloacal region, we previously reported that the *gar-3B* promoter is expressed in PCA, PCB, SPC and the spicule muscles; however, in *rg*Ex523, expression in PCA and the spicule muscles were extremely variable, but expression in PCB and SPC were consistent. The mosaic nature of *rg*Ex523 does not affect the experiments, since PCA, PCB and SPC are highly wired together. Stimulation of any one would result in increased activity of the set. The males were then put on an Olympus IX81microscope scope fitted with the Mosaic illumination targeting system (Andor Technology, USA). Using the Metamorph microscopy automation and imaging analysis software (Molecular Devices, PA), illumination regions were specified over the areas of the cloacal ganglia and dopaminergic ray neurons. The software then controlled the Mosaic targeted illumination system mirrors to reflect the filtered blue and green excitation light to the G-CaMP3/mDsRed expressing dopaminergic rays for ∼4.2 sec, followed by directing the illumination to both the ChR2-expressing cloacal ganglia and G-CaMP3-expressing dopaminergic rays for ∼5.7 sec, and then redirect the illumination to only the dopaminergic rays for ∼4.2 sec. The time between illumination protocols varied from 0.1 to 0.5 sec. The G-CaMP and mDsRed fluorescence signals were recorded simultaneously using an Optosplit II simultaneous image splitter (Cairn Research, UK) and an Andor iXon EM CCD camera, at the speed of ∼35 frames per second. After the males were recorded, the coverslip of the immobilized male was removed. If the male did not immediately crawl around the slide, the data was discarded. The fluorescence data was analyzed using the SimplePCI software and Microsoft Excel, as described earlier.

For ChR2 activation of ray neurons, L4 *dop-2; dop-3*; *rg*Ex550[*Pdat-1*:Chr2::YFP] and heterozygous males were incubated overnight with +/− all-*trans*-retinal. The adult males were then placed in a 2% noble agar pad containing 5 mM ARE on a slide and covered with a cover slip, while simultaneously being exposed to 4.2 mW/mm^2^ blue light illuminated through a 10× objective fitted to a Zeiss Stemi SV 11 stereomicroscope. To determine the working ARE concentration in 2% agar pads, a dose response curved was done with wild-type males (Figure S1C).

### Spicule prodding rate measurements

The spicule movements of wild-type or *dop-2(lf); dop-3(lf)* males copulating with heat-shocked paralyzed *pha-1*; *lite-1; him-5; rg*Ex431[P*hsp-16*:*egl-2(n693gf)*cDNA; P*unc-103E*:mDsRed; *pha-1(+)*] hermaphrodites were digitally recorded with Hamamatsu ImagEM camera at a rate of ∼35 frames per second. The grey-scale recordings were analyzed using the SimplePCI software. The recordings were reviewed to find the first instance where the male repetitively prods the vulva with his spicules for an uninterrupted duration of 6 to 10 seconds. In those frames of the recording, a rectangular ROI was drawn over the region of the male spicule. In the ROI of each frame, the standard deviation of the mean pixel intensity was calculated. The data was transferred to Microsoft Excel and plotted against time. Oscillation amplitudes greater than 5% were considered to be due to a spicule deflection. The durations between oscillations were graphed in Figure 5C.

### Length measurements

The In-Contact Length (ICL) was calculated by using the SimplePCI imaging software skeletonized tool to measure the length of the male outline that contacted the hermaphrodite cuticle of a representative frame. This measurement was then divided by the total male body length and converted to percentage values.

### RNAi

To monitor *gpa-16* RNAi effectiveness, pPC41[P*gpa-16*:*gpa-16*::YFP] was injected into *goa-1(lf); gpa-7(lf)* strain. RNAi was induced by feeding worms bacteria producing double stranded RNA (dsRNA) to the target *gpa-16* ORF. Bacteria with the L4440 double-T7 vector including *gpa-16* fourth and fifth exons were grown and induced by IPTG using a standard protocol [67]. L4 males expressing the pPC41 transgene were transferred to plates spotted with the dsRNA bacteria or OP50, as a control, and incubated for 20 hours. In a subset of these males, fluorescence of pharyngeal muscles and PDE neurons was checked. We found a similar percentage of males glowing in both tissues when fed with OP50 (80% vs. 75%, n = 20); however when males were fed with dsRNA there was a reduction in fluorescence of pharyngeal muscles when compared to PDE (78% vs. 4%, n = 23). The adult males then were assayed for their response to DA+ARE drug baths (Table 2).

## Supporting Information

Figure S1 Male mating sub-steps and drug test controls. (A) Representative frames taken from recorded behavioral movies for each step of mating. (B) Percentage of paralyzed males when treated with 20 mM of DA and water (n = 30 for each data set). (C) Dose response curve for spicule protraction in 2% agarose ARE pads (n = 30 for each data set).
(TIF)

Figure S2 Mating profiles of DA deficient males, related to Figure 1. (A) The duration wild type (n = 15) and *cat-2(lf)* (n = 21) males require to contact a paralyzed hermaphrodite in a 10 min observation. (B–C) Wild type (n = 20) and *cat-2(lf)* (n = 22) males mated with paralyzed hermaphrodites. (B) The time males spent in contact with a hermaphrodite's vulva and (C) the number of vulval contacts with a particular mate until insertion or in 120 sec. (D–F) *Pdat-1:G-CaMP3* (n = 16) and *Pdat-1:YFP* (n = 14) males mated with paralyzed hermaphrodites. (D) The duration in contact with a mate between insertion attempts. (E) The time males spent in contact with a hermaphrodite's vulva. (F) The number of vulval contacts with a particular mate until insertion or in 120 sec. Symbols represent an individual male performance. Open symbols represent unsuccessful insertions. Line and error bars represent mean and SEM.
(TIF)

Figure S3 Male tail expression of *dop-2*, *dop-3* and *gpa-7*, related to Figure 2. Post-cloacal sensilla B (PCB), dorsal spicule protractor (DSP), ventral spicule protractor (VSP), dorsal spicule retractor (DSR), and ventral spicule retractor (VSR). (A–G) DIC (right) and fluorescence (left) images of adult tail regions. (A–C) Expression patterns of P*dop-2*:YFP, (D&E) P*dop-3*:RFP, and (F&G) P*gpa-7*:YFP. Scale bar 10 µM.
(TIF)

Figure S4 Rays 5A, 7A, 9A Ca^+2^ transients during mating, related to Figure 3. The Ca^+2^ transients were determined by comparing G-CaMP and mDsRed intensity. mDSRed was used to normalize G-CaMP measurements to account for focus and illumination artifacts occurring while males mated with a paralyzed hermaphrodite at 10× magnification. (A) We measured the %ΔF/F0 of 7 wild type males during scanning for the hermaphrodite's vulva or attempting spicule insertion (prodding). The Y-axis depicts the %ΔF/F0 and X-axis the time scale. (B) We measured the %ΔF/F0 of 5 *Pdat-1:unc-103(gf)* males during scanning for the hermaphrodite's vulva or attempting spicule insertion (prodding). The In-Contact Length % (ICL%) are the numbers located at the top of each bar taken from a representative frame for each 1 sec intervals. The red arrow indicates exact time of vulval contact.
(TIF)

Figure S5 Sex-muscle Ca^+2^ transients during spicule insertion attempts, related to Figure 3. The Ca^+2^ transients determined by %ΔF/F0 (Y-axis) when at the vulva trying to insert their spicules during 10 secs (X-axis). For each subset of cells measured at 20× magnification, five different males are shown. For posterior sex-muscles: gubernaculum erector, anal depressor and ventral protractor expressed the P*unc-103E*:G-CaMP.
(TIF)

Figure S6 Ca^+2^ transients changes in Rn7A in individual males, related to Figure 4C. The %ΔF/F0 representing Rn7A neuron Ca^+2^ transients before, during and after PCB, SPC stimulation, for individual males grown on all *trans* retinal. The average and standard deviation of these traces are shown in Figure 4C. The boxed region denotes when blue excitation light was applied to the region of SPC and PCB. In some males, a slow increase of Rn7A fluorescence occurred during sec 0.6 to 4.2 due to stimulation of SPC/PCB from low intensity stray illumination of the Rn5A, Rn7A and Rn9A neurons. (Bottom- Middle), cartoon depicting the general area of illumination (blue ovals) of DA ray neurons and SPC PCB cloacal ganglia neurons (green circles).
(TIF)

Figure S7 Mating profiles of D2-like signaling deficient males, related to Figure 5. (A–H) wild type (n = 49) , *dop-2(lf)* (n = 18), *dop-3(lf)* (n = 14) and *dop-2(lf); dop-3(lf)* (n = 20) males were mated into paralyzed hermaphrodites and mating performance, until insertion or 120 sec, was assayed. (A–C) Time in contact with the hermaphrodite cuticle between vulva insertion attempts. (D&E) Time males spent in contact with the vulva during insertion attempts. (F–H) Number of vulval contacts with a hermaphrodite. Symbols represent an individual male performance. Open symbols represent unsuccessful insertions. Line and error bars represent mean and SEM.
(TIF)

Figure S8 Frequency of spicule thrusts during spicule insertion attempts, related to Figure 5C. Temporal profiles of spicule thrusts during 6 seconds of spicule insertion attempts for individual males. Blue lines denote when the spicule retracts and then thrusts against the vulval slit. The intervals between the blue lines include the duration that the spicule depresses the vulval slit. The average and standard deviation of the spicule thrust frequency are listed above each profile. In Figure 5C, for each male the distribution of individual spicule thrust intervals were plotted.
(TIF)

Figure S9 Refractory period, contact frequency, duration in contact with unproductive mates, and number of *fog-2* females impregnated for D2-like signaling deficient males, related to Figure 6. (A) The refractory period between ejaculations of wild type (n = 10) and *dop-2(lf); dop-3(lf)* (n = 10) males after pairings with moving hermaphrodites. (B) Average time males spent in contact with either a female or a paralyzed male, calculated when pairing one wild type or *dop-2; dop-3(lf)* males, singly with a one *fog-2(lf)* female and 10 paralyzed males. (C) Number of transient contacts with either a *fog-2(lf)* female or a paralyzed male calculated when pairing one wild type or *dop-2; dop-3(lf)* males with a single *fog-2(lf)* female and 10 paralyzed males. Symbols represent an individual male performance. Open symbols represent unsuccessful insertions. (A–C) Line and error bars represent mean and SEM. (D) The time required for a *dop-2; dop-3* or a wild type male to contact another worm in a 1.5 cm diameter bacterial lawn containing 6 paralyzed males and 6 paralyzed hermaphrodites. Line and error bars represent mean and SD. (E) Number of *fog-2* females impregnated after 4 or 18 hrs when paired with a single *fog-2(lf)* female, *1 fog-2(lf)* and 10 *C. briggsae* hermaphrodites, and *1 fog-2(lf)* and 10 *C. remanei* females. (F) The average time in contact with males that a wild type and *dop-2; dop-3* male, represented by each symbol, spent when surrounded by 40–50 paralyzed males. Each data subset depicts non-ablated animals (mock), PCB and p.c.s. ablated animals. *p-values* calculated using the Mann-Whitney test. Line and error bars represent mean and SEM.
(TIF)

Figure S10 (A) The cumulative distance traveled in one minute by 9 individual wild type or *dop-2; dop-3* males. (B) The velocities plotted respect to time of the 9 individual wild type and *dop-2; dop-3* males depicted in (A).
(TIF)

Figure S11 (A) Representative video montage of Ca^+2^ imaging recorded during mating. Each red square represents individual ROI's. ROI 1&3 indicate red and green backgrounds respectively; 2&4 indicate ray neuron fluorescence for G-CaMP3 and mDSred respectively. (B–F) The mean pixel intensity in arbitrary units (AU) shown on the Y-axis plotted against time (sec). (B) Raw mean pixel intensities determined from ROI's of the green channel. (C) Raw mean pixel intensities determined from ROI's of the red channel. (D) Green and red traces are the background fluorescence from each channel, subtracted from ray neuron G-CaMP3 and mDSred fluorescence, respectively. (E) The black solid line indicates the average normalized red value. (F) Green channel values corrected to the inverse of the red average values. (G) The %ΔF/F0 represents the percent flourescent changes from the last frame. The arbitrary F0 value is the fluorescence value in the first frame of the recordings.
(TIF)

Table S1 Acetylcholine receptor genes required for ARE-induced protraction.
(DOCX)

Table S2 Primers used in this study.
(DOCX)

Video S1 DA ray neurons activities increase during vulval spicule insertion attempts. We measured Ca^+2^ transients in Ray5A, Ray7A and Ray9A ray neurons during mating by comparing the fluorescence emissions of the G-CaMP3 Ca^+2^ sensor (green channel) and mDSred (red channel) co-expressed from the DA reuptake transporter promoter (P*dat-1*). The ray neurons are the most posterior cells in the male tail. We applied a pseudocolor spectrum to the images for better visualization. Blue = baseline fluorescence; Red = maximum fluorescence.
(WMV)

Video S2 Stimulation of cholinergic spicule neurons increases Ca^+2^ transients in Ray7A. We photo-stimulated PCB and SPC neurons expressing channelrhodopsin-2 (ChR2), a light sensitive cation channel, while simultaneously recording the G-CaMP3 fluorescence in ray neurons. The PCB and SPC neurons are stimulated from 4.5 secs until 10.5 secs. G-CaMP3 Ca^+2^ sensor (green channel) and mDSred (red channel). We applied a pseudocolor spectrum to the images for better visualization. Blue = baseline fluorescence; Red = maximum fluorescence.
(WMV)
