# Supplementary figures and images for: C. elegans Dopaminergic D2-Like Receptors Delimit Recurrent Cholinergic-Mediated Motor Programs during a Goal-Oriented Behavior
Source: PLoS Genet. 2012 Nov 15;8(11):e1003015. doi: 10.1371/journal.pgen.1003015 (PMC3499252; doi:10.1371/journal.pgen.1003015)

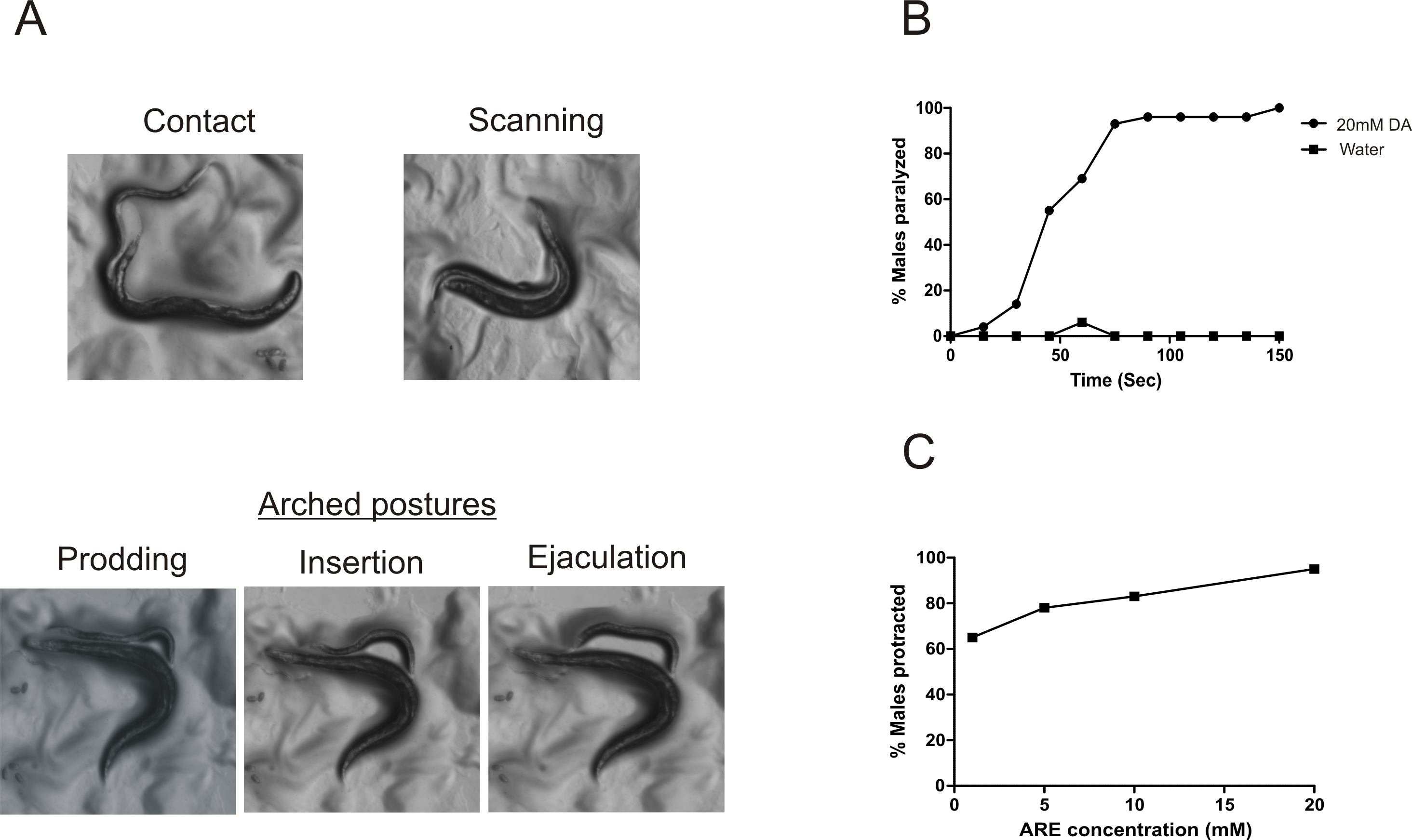

Supplement: Figure S1 — Male mating sub-steps and drug test controls. (A) Representative frames taken from recorded behavioral movies for each step of mating. (B) Percentage of paralyzed males when treated with 20 mM of DA and water (n = 30 for each data set). (C) Dose response curve for spicule protraction in 2% agarose ARE pads (n = 30 for each data set). (TIF) [file pgen.1003015.s001.tif]

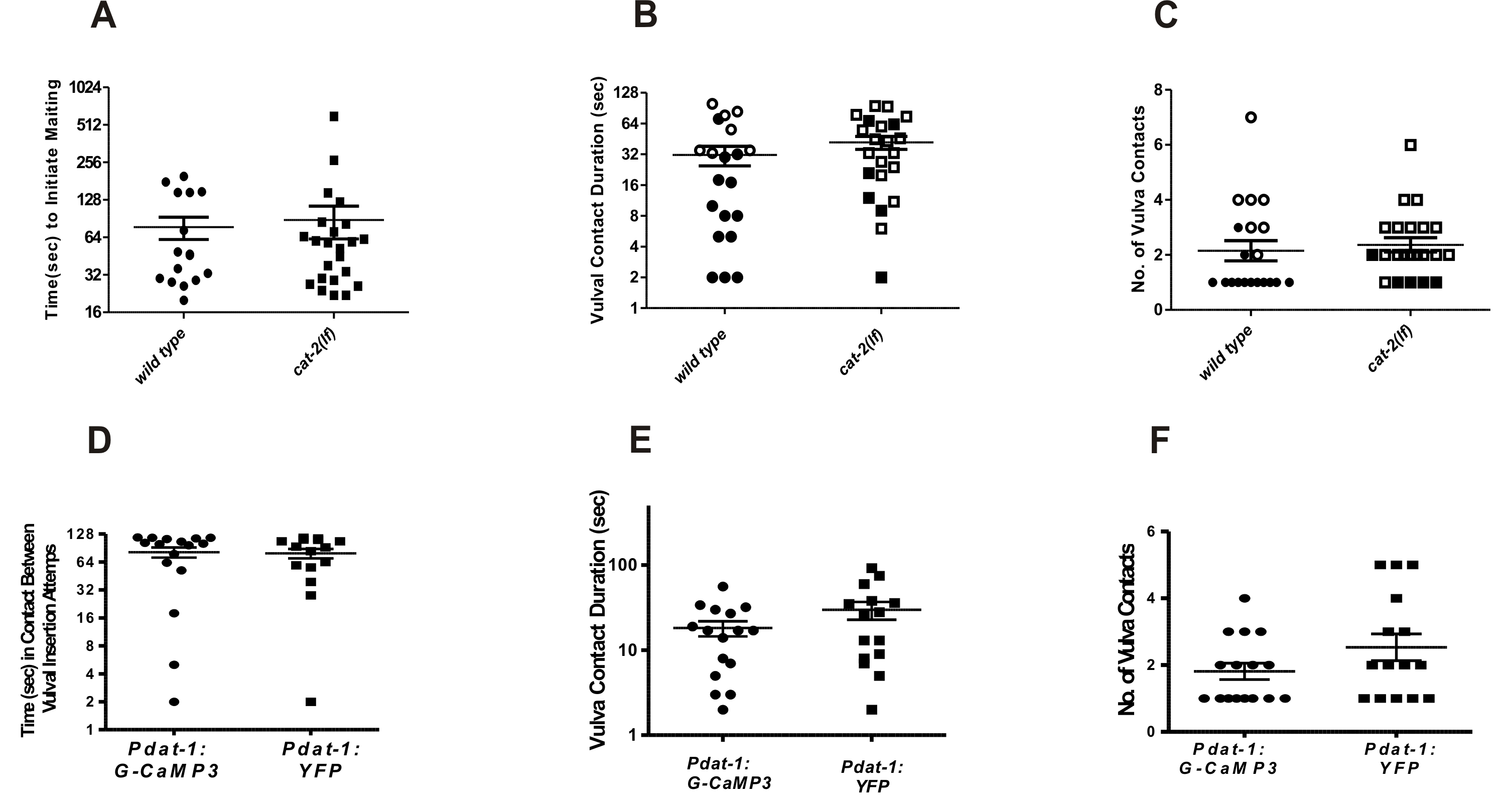

Supplement: Figure S2 — Mating profiles of DA deficient males, related to Figure 1. (A) The duration wild type (n = 15) and cat-2(lf) (n = 21) males require to contact a paralyzed hermaphrodite in a 10 min observation. (B–C) Wild type (n = 20) and cat-2(lf) (n = 22) males mated with paralyzed hermaphrodites. (B) The time males spent in contact with a hermaphrodite's vulva and (C) the number of vulval contacts with a particular mate until insertion or in 120 sec. (D–F) Pdat-1:G-CaMP3 (n = 16) and Pdat-1:YFP (n = 14) males mated with paralyzed hermaphrodites. (D) The duration in contact with a mate between insertion attempts. (E) The time males spent in contact with a hermaphrodite's vulva. (F) The number of vulval contacts with a particular mate until insertion or in 120 sec. Symbols represent an individual male performance. Open symbols represent unsuccessful insertions. Line and error bars represent mean and SEM. (TIF) [file pgen.1003015.s002.tif]

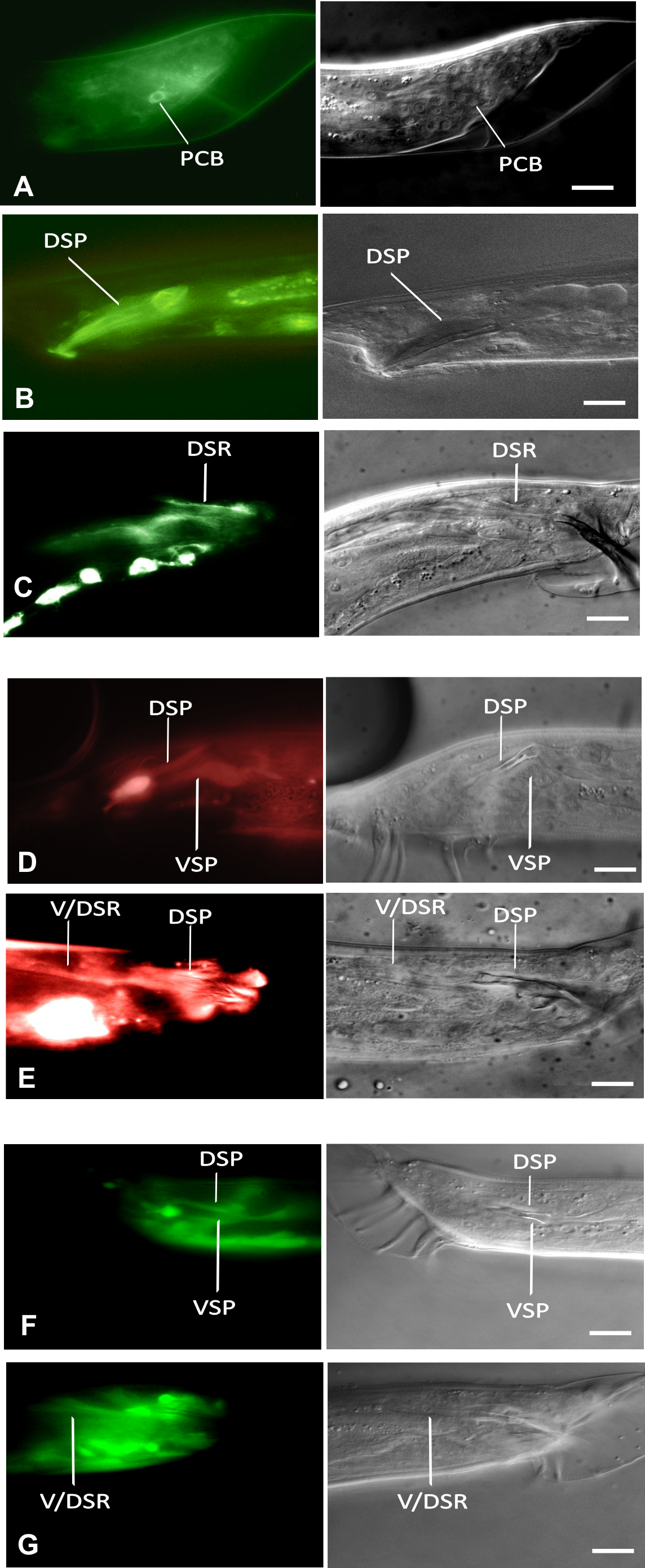

Supplement: Figure S3 — Male tail expression of dop-2, dop-3 and gpa-7, related to Figure 2. Post-cloacal sensilla B (PCB), dorsal spicule protractor (DSP), ventral spicule protractor (VSP), dorsal spicule retractor (DSR), and ventral spicule retractor (VSR). (A–G) DIC (right) and fluorescence (left) images of adult tail regions. (A–C) Expression patterns of Pdop-2:YFP, (D&E) Pdop-3:RFP, and (F&G) Pgpa-7:YFP. Scale bar 10 µM. (TIF) [file pgen.1003015.s003.tif]

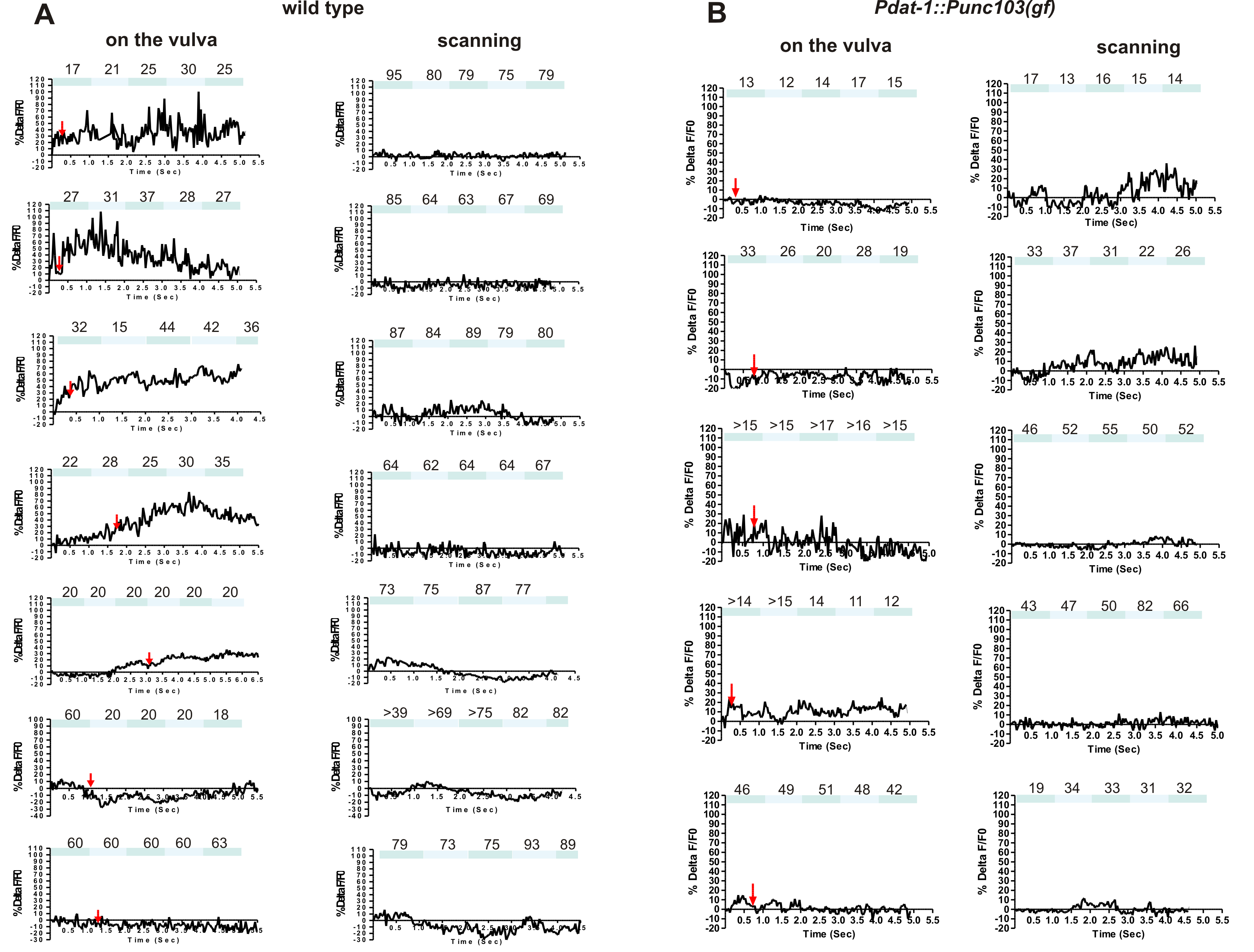

Supplement: Figure S4 — Rays 5A, 7A, 9A Ca+2 transients during mating, related to Figure 3. The Ca+2 transients were determined by comparing G-CaMP and mDsRed intensity. mDSRed was used to normalize G-CaMP measurements to account for focus and illumination artifacts occurring while males mated with a paralyzed hermaphrodite at 10× magnification. (A) We measured the %ΔF/F0 of 7 wild type males during scanning for the hermaphrodite's vulva or attempting spicule insertion (prodding). The Y-axis depicts the %ΔF/F0 and X-axis the time scale. (B) We measured the %ΔF/F0 of 5 Pdat-1:unc-103(gf) males during scanning for the hermaphrodite's vulva or attempting spicule insertion (prodding). The In-Contact Length % (ICL%) are the numbers located at the top of each bar taken from a representative frame for each 1 sec intervals. The red arrow indicates exact time of vulval contact. (TIF) [file pgen.1003015.s004.tif]

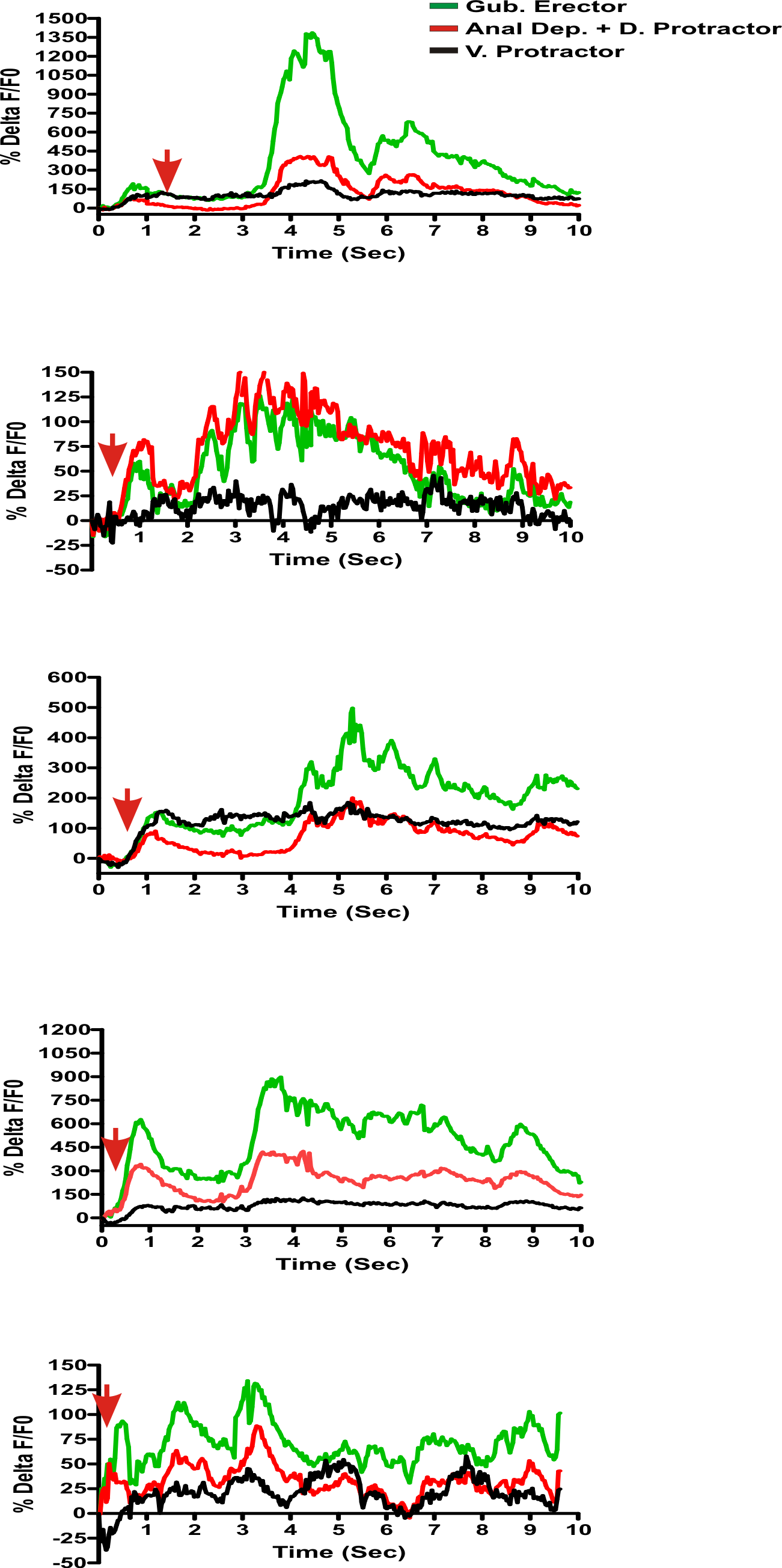

Supplement: Figure S5 — Sex-muscle Ca+2 transients during spicule insertion attempts, related to Figure 3. The Ca+2 transients determined by %ΔF/F0 (Y-axis) when at the vulva trying to insert their spicules during 10 secs (X-axis). For each subset of cells measured at 20× magnification, five different males are shown. For posterior sex-muscles: gubernaculum erector, anal depressor and ventral protractor expressed the Punc-103E:G-CaMP. (TIF) [file pgen.1003015.s005.tif]

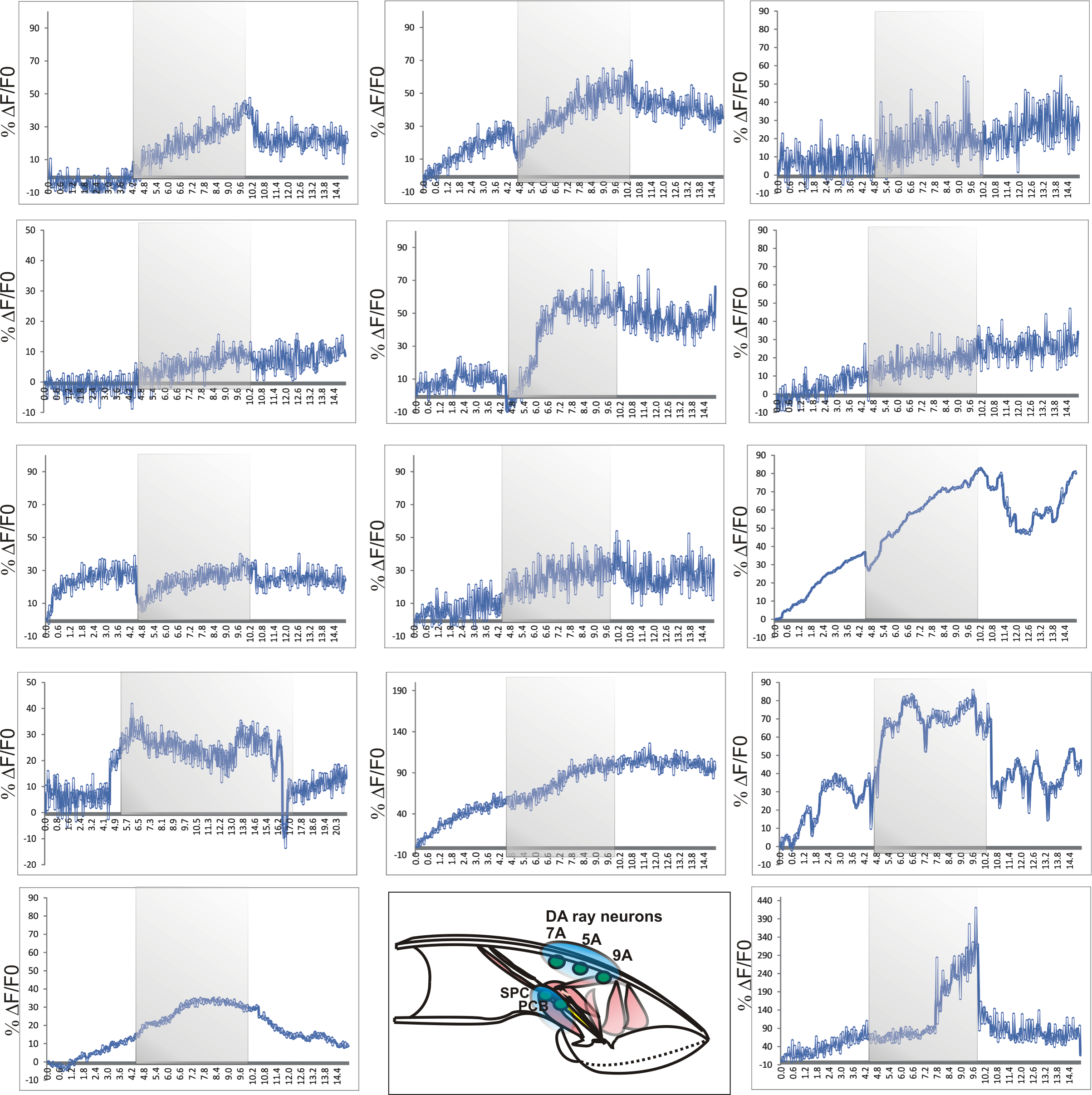

Supplement: Figure S6 — Ca+2 transients changes in Rn7A in individual males, related to Figure 4C. The %ΔF/F0 representing Rn7A neuron Ca+2 transients before, during and after PCB, SPC stimulation, for individual males grown on all trans retinal. The average and standard deviation of these traces are shown in Figure 4C. The boxed region denotes when blue excitation light was applied to the region of SPC and PCB. In some males, a slow increase of Rn7A fluorescence occurred during sec 0.6 to 4.2 due to stimulation of SPC/PCB from low intensity stray illumination of the Rn5A, Rn7A and Rn9A neurons. (Bottom- Middle), cartoon depicting the general area of illumination (blue ovals) of DA ray neurons and SPC PCB cloacal ganglia neurons (green circles). (TIF) [file pgen.1003015.s006.tif]

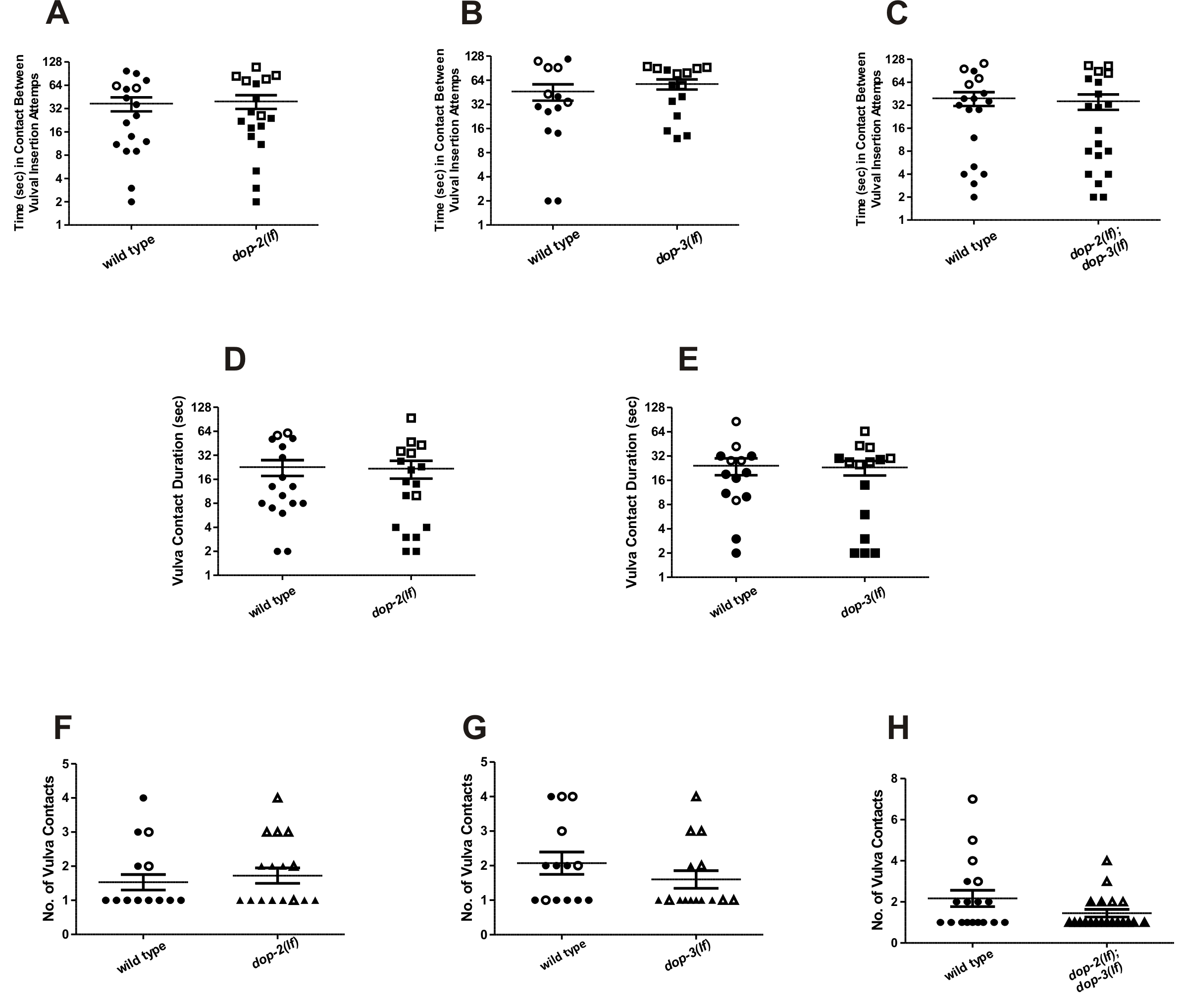

Supplement: Figure S7 — Mating profiles of D2-like signaling deficient males, related to Figure 5. (A–H) wild type (n = 49) , dop-2(lf) (n = 18), dop-3(lf) (n = 14) and dop-2(lf); dop-3(lf) (n = 20) males were mated into paralyzed hermaphrodites and mating performance, until insertion or 120 sec, was assayed. (A–C) Time in contact with the hermaphrodite cuticle between vulva insertion attempts. (D&E) Time males spent in contact with the vulva during insertion attempts. (F–H) Number of vulval contacts with a hermaphrodite. Symbols represent an individual male performance. Open symbols represent unsuccessful insertions. Line and error bars represent mean and SEM. (TIF) [file pgen.1003015.s007.tif]

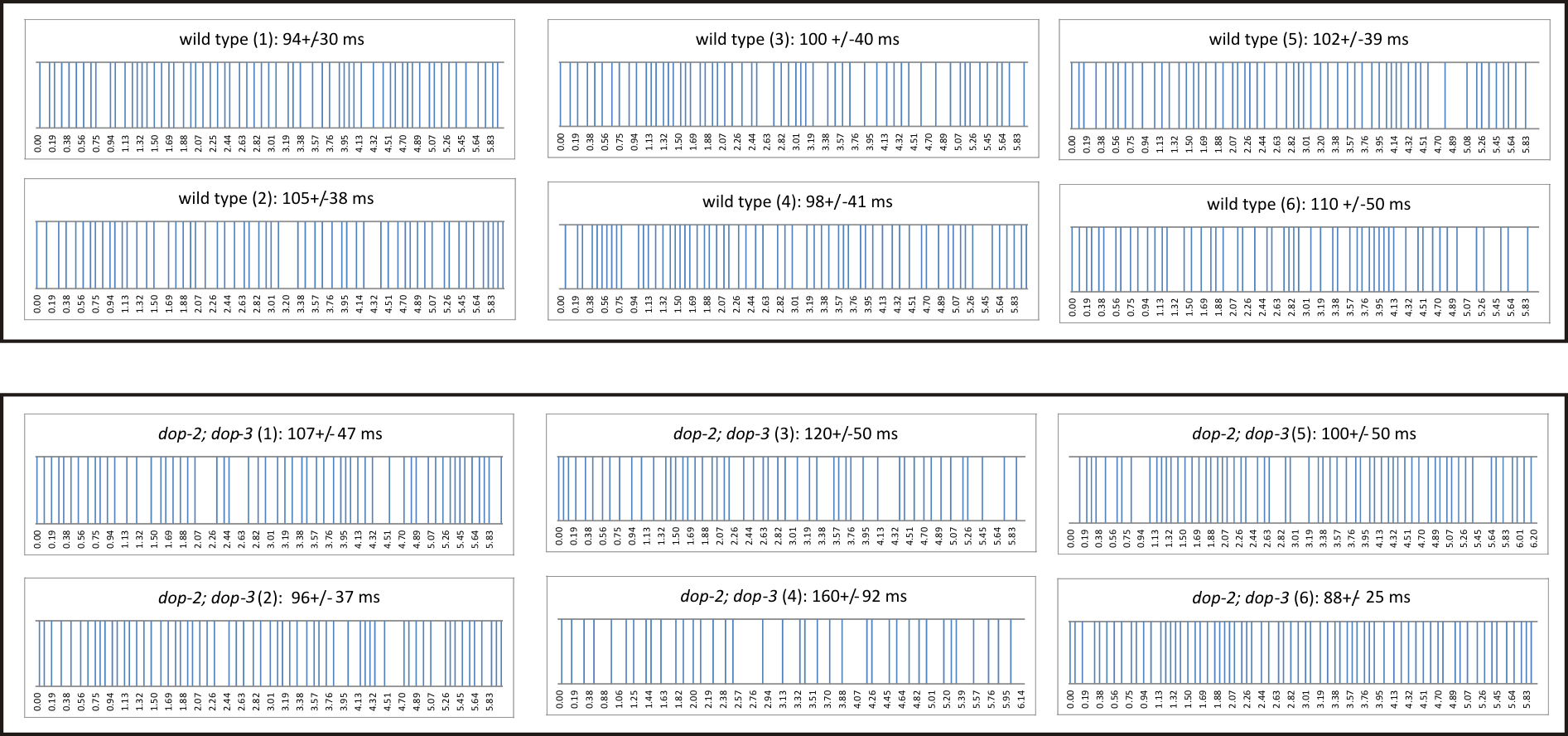

Supplement: Figure S8 — Frequency of spicule thrusts during spicule insertion attempts, related to Figure 5C. Temporal profiles of spicule thrusts during 6 seconds of spicule insertion attempts for individual males. Blue lines denote when the spicule retracts and then thrusts against the vulval slit. The intervals between the blue lines include the duration that the spicule depresses the vulval slit. The average and standard deviation of the spicule thrust frequency are listed above each profile. In Figure 5C, for each male the distribution of individual spicule thrust intervals were plotted. (TIF) [file pgen.1003015.s008.tif]

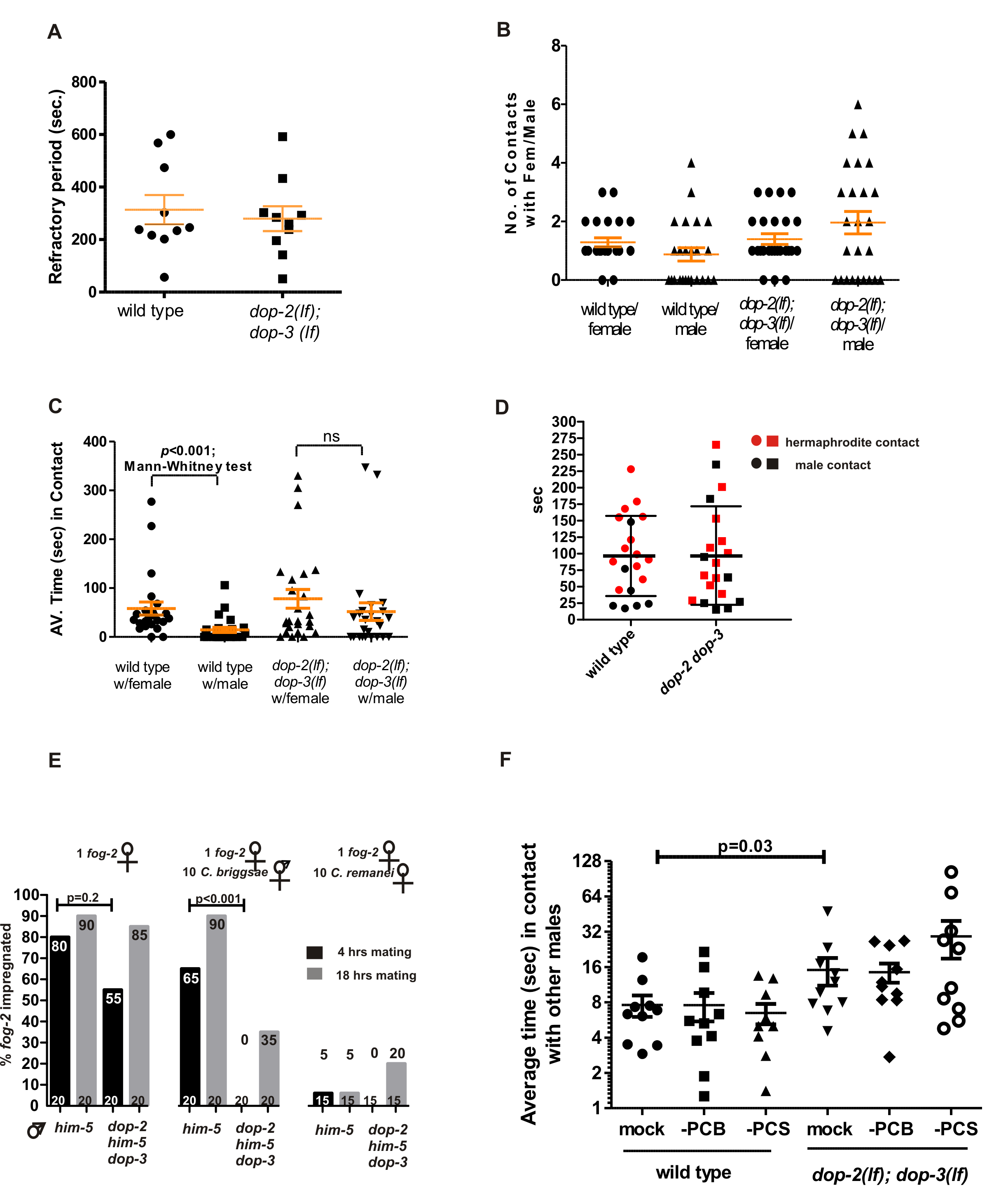

Supplement: Figure S9 — Refractory period, contact frequency, duration in contact with unproductive mates, and number of fog-2 females impregnated for D2-like signaling deficient males, related to Figure 6. (A) The refractory period between ejaculations of wild type (n = 10) and dop-2(lf); dop-3(lf) (n = 10) males after pairings with moving hermaphrodites. (B) Average time males spent in contact with either a female or a paralyzed male, calculated when pairing one wild type or dop-2; dop-3(lf) males, singly with a one fog-2(lf) female and 10 paralyzed males. (C) Number of transient contacts with either a fog-2(lf) female or a paralyzed male calculated when pairing one wild type or dop-2; dop-3(lf) males with a single fog-2(lf) female and 10 paralyzed males. Symbols represent an individual male performance. Open symbols represent unsuccessful insertions. (A–C) Line and error bars represent mean and SEM. (D) The time required for a dop-2; dop-3 or a wild type male to contact another worm in a 1.5 cm diameter bacterial lawn containing 6 paralyzed males and 6 paralyzed hermaphrodites. Line and error bars represent mean and SD. (E) Number of fog-2 females impregnated after 4 or 18 hrs when paired with a single fog-2(lf) female, 1 fog-2(lf) and 10 C. briggsae hermaphrodites, and 1 fog-2(lf) and 10 C. remanei females. (F) The average time in contact with males that a wild type and dop-2; dop-3 male, represented by each symbol, spent when surrounded by 40–50 paralyzed males. Each data subset depicts non-ablated animals (mock), PCB and p.c.s. ablated animals. p-values calculated using the Mann-Whitney test. Line and error bars represent mean and SEM. (TIF) [file pgen.1003015.s009.tif]

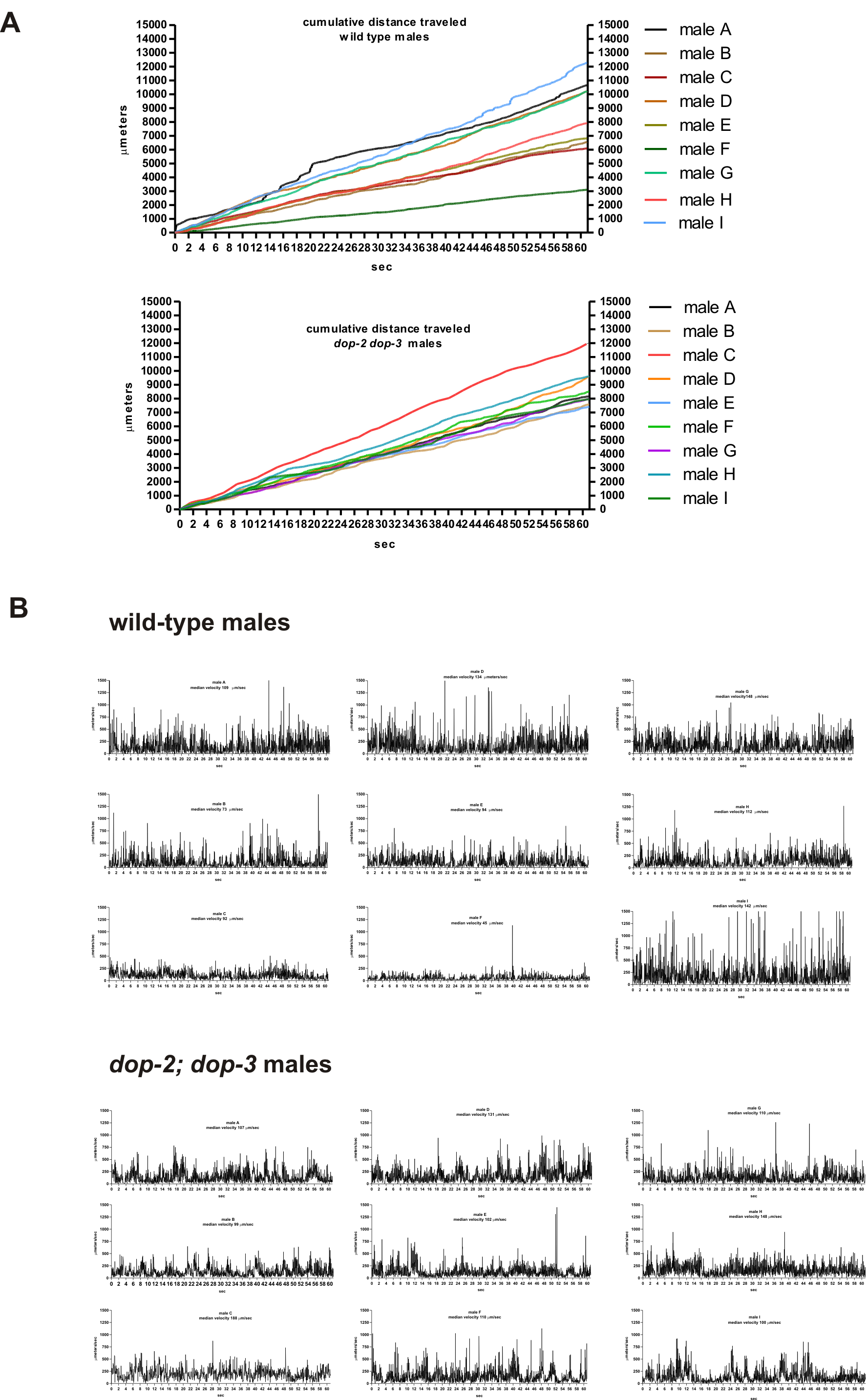

Supplement: Figure S10 — (A) The cumulative distance traveled in one minute by 9 individual wild type or dop-2; dop-3 males. (B) The velocities plotted respect to time of the 9 individual wild type and dop-2; dop-3 males depicted in (A). (TIF) [file pgen.1003015.s010.tif]

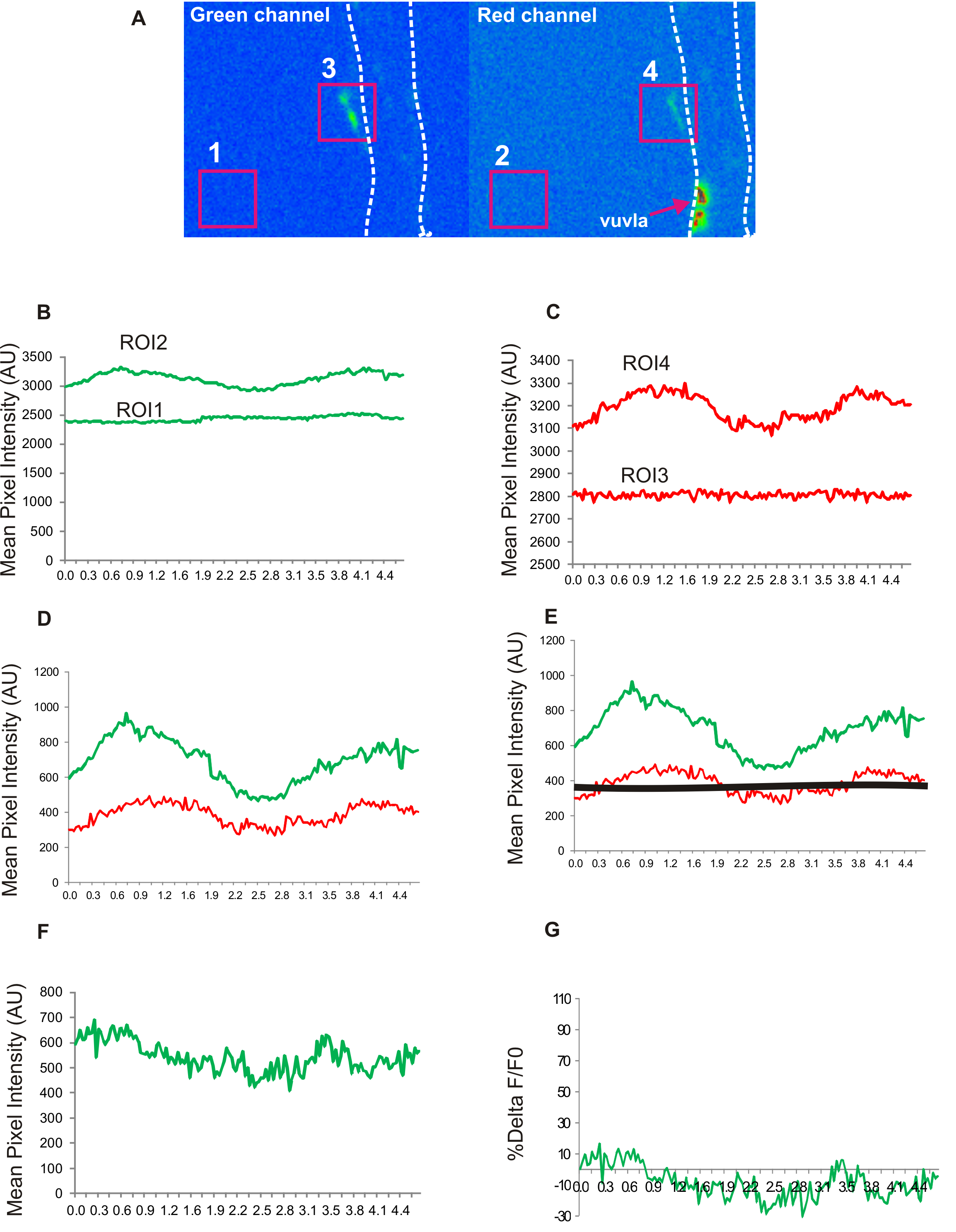

Supplement: Figure S11 — (A) Representative video montage of Ca+2 imaging recorded during mating. Each red square represents individual ROI's. ROI 1&3 indicate red and green backgrounds respectively; 2&4 indicate ray neuron fluorescence for G-CaMP3 and mDSred respectively. (B–F) The mean pixel intensity in arbitrary units (AU) shown on the Y-axis plotted against time (sec). (B) Raw mean pixel intensities determined from ROI's of the green channel. (C) Raw mean pixel intensities determined from ROI's of the red channel. (D) Green and red traces are the background fluorescence from each channel, subtracted from ray neuron G-CaMP3 and mDSred fluorescence, respectively. (E) The black solid line indicates the average normalized red value. (F) Green channel values corrected to the inverse of the red average values. (G) The %ΔF/F0 represents the percent flourescent changes from the last frame. The arbitrary F0 value is the fluorescence value in the first frame of the recordings. (TIF) [file pgen.1003015.s011.tif]
