# Supplementary material for: C. elegans Dopaminergic D2-Like Receptors Delimit Recurrent Cholinergic-Mediated Motor Programs during a Goal-Oriented Behavior
Source: PLoS Genet. 2012 Nov 15;8(11):e1003015. doi: 10.1371/journal.pgen.1003015 (PMC3499252; doi:10.1371/journal.pgen.1003015)
Supplement: Table S1 — Acetylcholine receptor genes required for ARE-induced protraction. (DOCX) [file pgen.1003015.s012.docx]

**TableS1.** Acetylcholine receptor genes required for ARE-induced protraction

| **Genotype** | **% Males protracted (n)**  **ARE concentration** | | | | | |
| --- | --- | --- | --- | --- | --- | --- |
|  | **50mM** | **10mM** | **1mM** | **100µM** | **10µM** | **EC_50_** |
| Wild type | ND | 98 (17) | 87 (30) | 64 (25) | 11 (20) | 50µM |
| *unc-29*  *(e193)* | ND | 100 (19) | 95 (30) | 58 (24) | 11 (18) | 71µM |
| *gar-3*  *(gk305)* | 100 (30) | 100 (29) | 81 (38) | 44 (38) | 18 (33) | 226 µM |
| *acr-16*  *(ok789)* | 100 (19) | 76 (32) | 60 (40) | 18 (32) | 23 (30) | 1mM |
| *gar-3; unc-29* | 93 (30) | 90 (30) | 83 (30) | 36 (30) | 3 (30) | 138 µM |
| *gar-3; acr-16* | 93 (30) | 76 (30) | 46 (30) | 23 (30) | 3 (30) | 1.2mM |
| *unc-29;*  *acr-16* | 10 (30) | 10 (30) | ND | ND | 5 (30) | >50mM |
| *acr-16;*  *unc-29;*  *gar-3* | 13 (30) | 4 (23) | ND | ND | 5 (15) | >50mM |
