# Supplementary material for: C. elegans Dopaminergic D2-Like Receptors Delimit Recurrent Cholinergic-Mediated Motor Programs during a Goal-Oriented Behavior
Source: PLoS Genet. 2012 Nov 15;8(11):e1003015. doi: 10.1371/journal.pgen.1003015 (PMC3499252; doi:10.1371/journal.pgen.1003015)
Supplement: Table S2 — Primers used in this study. (DOCX) [file pgen.1003015.s013.docx]

**TableS2.** Primers used in this study

| **Primer name** | **Sequence** |  |
| --- | --- | --- |
| DOP3geneF | gcgcccggatccatgttggctggacaacaccacgttacagac |  |
| DOP3geneR | gcgcccaccggttctttttttgaatatcccgcatgataaaatgttccggaag |  |
| dop3(vs106)F | cgaaattcaaatcttcttccatcttcttgc |  |
| dop3(vs106)R | gtctataaaaagcgatgtgggcac |  |
| FDop2 | atggaggccggagagacatgg |  |
| Dop2R | atggcataactatgatggccac |  |
| ATTB1Dop2pr | ggggacaagtttgtacaaaaaagcaggctccgatttgtgctcacactcgtcgagtaccc |  |
| Attb2Dop2pr | ggggaccactttgtacaagaaagctgggtgtctctccggcctccagtttttggagttgg |  |
| RNAigpa16 | ggggacaagtttgtacaaaaaagcaggctcccgtgcaccaaggataatttatttgggcgc |  |
| Pgpa16Rv2 | ggg gaccactttgtacaagaaagctggtactcttcagtcgtatatccgacgtcgtg |  |
| Pgpa16Fv2 | ggggaccactttgtacaagaaagctgggtactcttcagtcgtatatccgacgtcgtg |  |
| gpa7pk610indelR | ctagaaaatatggatagctccgttgactatgc |  |
| dop4(tm1392)F | ttggcttacgggtctgatccgaacg |  |
| dop4(tm1392)R | gcagaccaattttgtccaaccaccatcc |  |
| attb1DOP2F ggggacaagtttgtacaaaaaagcaggctatggaggccggagagacatggaatgtctc | |  |
| attb2DOP2R | ggggaccactttgtacaagaaagctgggtttagacatgcgcctgcttgttactgaaatgg |  |
| gpa7pk610F | gatgttggatcggttgcttttagcctgtc |  |
| gpa7pk610R | aagcgaatgatatcacttaccaccggg |  |
| gpa14pk347F | cttggaggacctttaagtggaaagagtac |  |
| gpa14pk347R | gctttaaatacactttccatgcaggcgcg |  |
| gpa14pkindelF | acgtgggtggtcaaagatcggaacgaa |  |
| Pgpa-7F | ggggacaagtttgtacaaaaaagcaggctccgacaactttctgccggacactgaccgttt |  |
| Pgpa-7R | ggggaccactttgtacaagaaagctgggtgatgatccgaagactcatcgattgatccttc |  |
|  |  |  |
